# Identification of Prognostic Metabolism-Related Genes in Clear Cell Renal Cell Carcinoma

**DOI:** 10.1155/2021/2042114

**Published:** 2021-09-27

**Authors:** Yusa Chen, Yumei Liang, Ying Chen, Shaxi Ouyang, Kanghan Liu, Wei Yin

**Affiliations:** Department of Nephrology, Hunan Provincial People's Hospital, The First Affiliated Hospital of Hunan Normal University, Changsha 410000, China

## Abstract

**Background:**

Clear cell renal cell carcinoma (ccRCC) is a cancer with abnormal metabolism. The purpose of this study was to investigate the effect of metabolism-related genes on the prognosis of ccRCC patients.

**Methods:**

The data of ccRCC patients were downloaded from the TCGA and the GEO databases and clustered using the nonnegative matrix factorization method. The limma software package was used to analyze differences in gene expression. A random forest model was used to screen for important genes. A novel Riskscore model was established using multivariate regression. The model was evaluated based on the metabolic pathway, immune infiltration, immune checkpoint, and clinical characteristics.

**Results:**

According to metabolism-related genes, kidney clear cell carcinoma (KIRC) datasets downloaded from TCGA were clustered into two groups and showed significant differences in prognosis and immune infiltration. There were 667 differentially expressed genes between the two clusters, of which 408 were screened by univariate analysis. Finally, 12 differentially expressed genes (*MDK*, *SLC1A1*, *SGCB*, *C4orf3*, *MALAT1*, *PILRB*, *IGHG1*, *FZD1*, *IFITM1*, *MUC20*, *KRT80*, and *SALL1*) were filtered out using the random forest model. The model of Riskscore was obtained by multiplying the expression levels of these 12 genes with the corresponding coefficients of the multivariate regression. We found that the Riskscore correlated with the expression of these 12 genes; the high Riskscore matched the low survival rate verified in the verification set. The analysis found that the Riskscore model was associated with most of the metabolic processes, immune infiltration of cells such as plasma cells, immune checkpoints such as PD-1, and clinical characteristics such as M stage.

**Conclusion:**

We established a new Riskscore model for the prognosis of ccRCC based on metabolism. The genes in the model provided several novel targets for the study of ccRCC.

## 1. Introduction

Approximately four hundred thousand people are diagnosed with renal cell carcinoma (RCC) worldwide every year. Approximately 70% of these patients have clear cell RCC (ccRCC) [1], one of the most common malignancies of the urinary system [2]. Genetically, the continuous loss of multiple tumor suppressor genes leads to ccRCC [3]. Surgical resection is the main treatment for early-stage ccRCC, but approximately three out of ten patients have metastasis after resection [4]. ccRCC is not sensitive to radiotherapy and chemotherapy [5]; therefore, more effective targeted therapy is needed. Other studies have explored the possible molecular markers or therapeutic targets of ccRCC from different perspectives, such as autophagy associated long noncoding RNAs (lncRNAs) [6], methylation modification of m6A [7], DNA methylation [8], and immune invasion [9]. We set out to find new potential targets from a metabolic perspective.

Mutations in cancer cells can lead to metabolic reprogramming causing abnormal metabolic patterns to meet the different needs from normal cells for cancer proliferation and growth [10]. Increased aerobic glycolysis and impaired oxidative phosphorylation, known as the Warburg effect [11], occur in cancer. The research and development of many anticancer drugs are aimed at the changes in these metabolic pathways [12]. The transformation of renal epithelial cells into ccRCC leads to a decrease in the level of fatty acid oxidation and damage to the mitochondrial structure; the resulting accumulation of glycogen and lipids is the cause of ccRCC transparency [13]. The upregulation of glycolysis pathway genes is a central event in the pathogenesis of ccRCC [14]. The research and development of many anticancer drugs are aimed at the changes in these metabolic pathways [12]. Further studies on the changes in metabolism in ccRCC are needed.

The random forest algorithm is a common machine-learning algorithm often used in cancer and biological research as a routine bioinformatics protocol; it was used to reduce the dimensions to screen more important genes. This method has been widely used in many different prognostic models of ccRCC such as chromatin-remodeling genes [15], DNA methylation patterns [16], and microRNA [17]. Cancer is usually accompanied by abnormal changes in metabolic patterns. Previous studies have also established prognostic models for healthy people and ccRCC patients from the perspective of metabolism [18]. Our study found some key gene differences between high-risk and low-risk ccRCC patients that were not previously noted by other researches, providing a new research target for a follow-up study.

It was recognized in as early as the 1960s that there is a relationship between immune infiltration and prognosis in different diseases [19]. The cells involved in immune infiltration are immune cells that appear in tumors and are divided into 22 types, such as T, B, NK, and plasma cells. For example, NK cells play a role in tumor immunity; receptor or coreceptor recognition of ligands on tumor cells can activate NK cells, resulting in targets with insufficient HLA I expression being killed [20]. The metabolism of NK cells is impaired in the tumor microenvironment [21]. In cancer, neutrophils may promote tumor progression, in part by producing reactive oxygen species (ROS) [22]. The rewiring of other metabolic pathways in neutrophils may affect their tumorigenesis/metastasis promoting function [23]; such pathways are the main components of nontumor constituents in the tumor microenvironment, and different types of malignant tumors often show different features of immune cell subsets [24]. The prognostic significance of T cell tumor infiltration has been widely accepted [25]. Immune checkpoints refer to the set of inhibitory pathways possessed by immune cells to regulate and control the durability of the immune response while maintaining self-tolerance [26]. Many successful immunotherapies targeting these checkpoints are already available to treat ccRCC [27].

In this study, by clustering the cancer genome atlas (TCGA) kidney clear cell carcinoma (KIRC) datasets according to metabolic patterns and screening differentially expressed genes of two clusters, we constructed and verified a Riskscore model and analyzed the relationships between Riskscore and metabolic pathway, immune infiltration, immune checkpoint, and clinical features.

## 2. Methods

### 2.1. Datasets and Preprocessing

The workflow of this study is presented in Supplementary Figure S1. The phenotype datasets and RNA sequencing datasets of TCGA Kidney Clear Cell Carcinoma (KIRC) were downloaded from UCSC Xena (https://xenabrowser.net/). Then, the number of fragments per kilobase million fragments (FPKM) was converted to transcript/value per million-word node (TPM). The microarray dataset GSE29609 (*n* = 30) was used as an external validation set accessed from the Gene Expression Omnibus (GEO; https://www.ncbi.nlm.nih.gov/geo/). Affymetrix was used to generate raw data of the microarray dataset and quantile normalization and background correction of these data were performed using the rapid motor adaptation algorithm in the affy package. The clinicopathologic features (gender, age, grade, stage, and status) of the TCGA KIRC dataset were sorted and are shown in Supplementary Table S1.

### 2.2. KIRC Metabolic Gene Clustered

The 2752 metabolism-related genes were obtained from a previous study [28]. A total of 2585 genes were identified in TCGA data. Then, 1416 genes were screened by a univariate cox, and KIRC data were classified by the nonnegative matrix factorization clustering method to determine the metabolism-related patterns; datasets of patients were clustered for further analysis.

### 2.3. Immune Infiltration and Pathway Analysis

The type and number of immune cells in KIRC samples were quantified using the cell-type identification by estimating relative subsets of known RNA transcripts algorithm [29] to compare the differences in immune infiltration in different cluster categories or risk groups. Gene set variation analysis was used to calculate the activity of the metabolic pathway using 114 metabolic pathway sets [29].

### 2.4. Establishment of the Metabolic Riskscore Model

The limma package was used to identify the genes related to metabolism (*P* < 0.05 and |logfc| > 1.5); univariate screening was performed, and random survival forest was used for further screening [30]. The Riskscore was the sum of the gene expression value ^*∗*^ regression coefficient (Riskscore = (0.1186^∗^*MDK*) + (−0.0505^∗^*SLC1A1*) + (−0.094^∗^*SGCB*) +(−0.1992^∗^*C4orf3*) + (0.1986^∗^*MALAT1*) + (0.1051^∗^*PILRB*)+ (0.0142^∗^*IGHG1*) + (−0.0541^∗^*FZD1*) + (0.2203^∗^*IFITM1*) + (−0.1682^∗^*MUC20*) + (0.1104^∗^*KRT80*) + (−0.2114^∗^*SALL1*)). The patients were divided into high-risk and low-risk groups using the surv_cutpoint method of the survminer package.

### 2.5. Cell Culture

A human ccRCC cell line (ZQ0339), CAKI-1, was cultured in McCoy's 5A medium (ZQ-1000). The cell line and the medium above were purchased from Shanghai Zhong Qiao Xin Zhou Biotechnology Co., Ltd. A normal human kidney proximal tubular cell line (CL-0109), HK-2, and its special medium (CM-0109) were purchased from Procell Life Science & Technology Co., Ltd. These cells were cultured at 5% CO_2_ and 37°C. The medium contained 10% FBS (Gibco) and 1% Penicillin-Streptomycin Solution (C0222, Beyotime).

### 2.6. Quantitative Real-Time PCR (qRT-PCR)

Relative RNA expressions of *SLC1A1*, *MALAT1, FZD1,* and *SALL1* in HK-2 and CAKI-1 cell lines were detected by qRT-PCR. The total RNA of cells was isolated by TRIzol^®^ (15596026, Thermo) and reverse-transcribed to cDNA by HiFiScript cDNA Synthesis Kit (CW2569, Cwbio). qPCR amplification was performed using UltraSYBR Mixture (CW2601, Cwbio) with QuantStudio™ 1 Real-Time PCR System (Thermo), and the cycling conditions were followed by the operating instructions. The sequences of the primers used in this study are shown in Table 1. The expression of *β*-actin was selected as an internal reference, and the relative RNA expression of genes was calculated by the 2^−ΔΔ^ CT method (the expression fold of genes in HK-2 was regarded as 1, respectively)

### 2.7. Statistical Analyses

Before the unpaired Student's *t*-test was used to compare the differences between the two groups, the Shapiro–Wilk test was used to detect whether the variables were normally distributed. If they did not conform to the normal distribution, the Wilcoxon test was used to compare the differences between the groups. Correlation coefficients were calculated using the Pearson correlation analysis and distance correlation analysis. The datasets of patients were divided into high-risk and low-risk groups based on dichotomy. The data were visualized using ggplot2 (a package for R). Survival curves of subgroups were generated by the Kaplan–Meier method. The statistical significance of differences in each dataset was identified using the log-rank test. Survival curves were generated by survminer (a package for R); heat maps were generated using pheatmap. All statistical analyses above were performed in the environment of R 3.6.1. All statistical tests were two-sided and considered statistically significant when the *P* value was <0.05. Column charts of relative RNA expressions were drawn by GraphPad Prism 8.0.2.

## 3. Results

### 3.1. Two Groups of Patients Clustered by Metabolic Patterns Had a Different Prognosis

**Figure 1:**
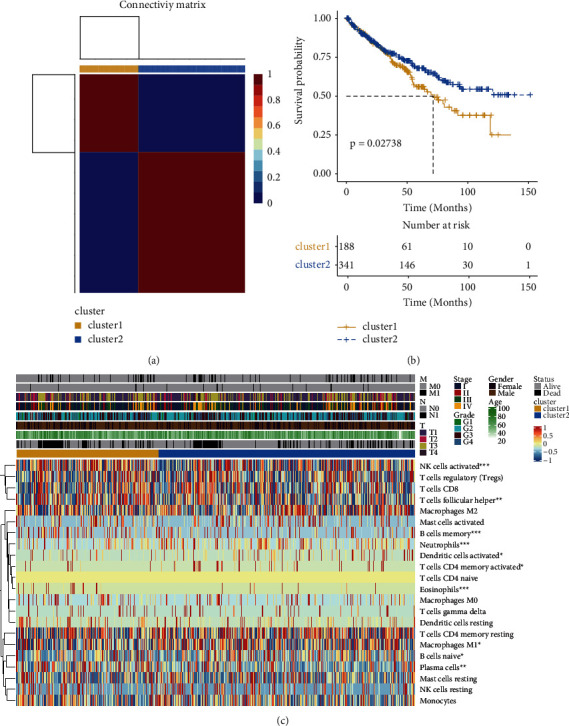
Clustering by metabolic genes. (a) Samples grouped into two categories. (b) Survival analysis of these two categories. (c) Distribution of samples and immune cells.

According to the metabolic genes, ccRCC patients were clustered into two groups (Figure 1(a)). It is suitable to divide the samples into 2 clusters rather than more clusters (Supplementary Figure S2). There were significant differences in the results of survival analysis between the two groups (Figure 1(b)). The distribution of immune cells in the two groups is shown in Figure 1(c). The results showed a significant difference between the two groups in NK cells activated, T cells follicular helper, B cells memory, neutrophils, dendritic cells activated, T cells CD4 memory activated, eosinophils, macrophages M1, B cells naive, and plasma cells. There was a significant difference in certainty between the two groups.

### 3.2. The Riskscore Model Was Established according to the Differentially Expressed Genes: the Higher the Score, the Worse the Prognosis

**Figure 2:**
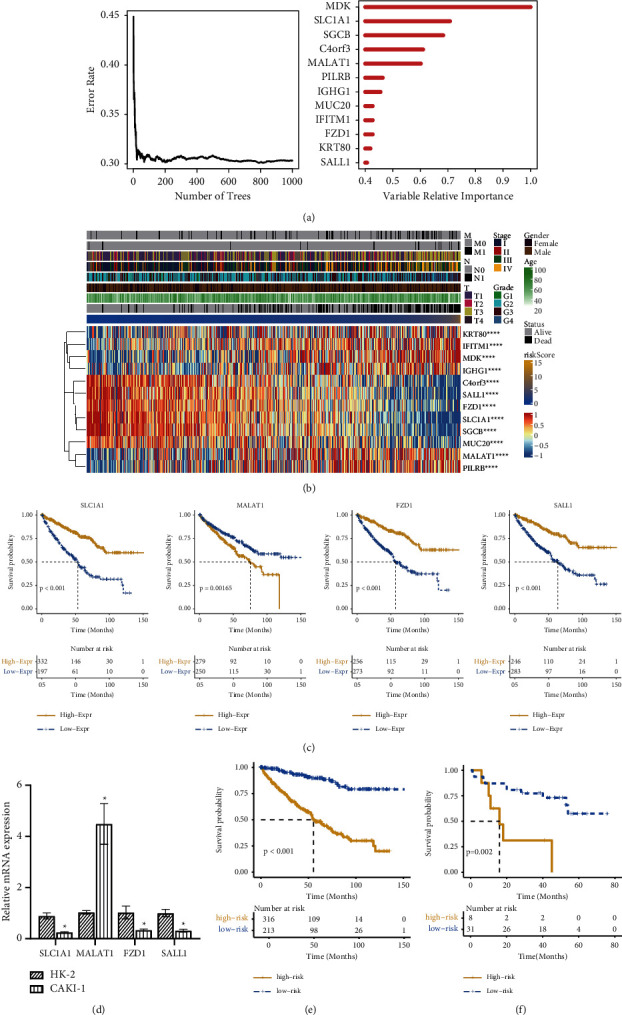
Establishment and verification of the prognosis score (Riskscore). (a) Error rate for the random trees and importance values for the 12 metabolism-related genes. (b) Riskscore modeling score, clinical characteristics, and specific expression of 12 genes. (c) Survival analysis of *FZD1*, *SALL1*, *SLC1A1*, and *MALAT1*. (d) Relative mRNA expressions of *FZD1*, *SALL1*, *SLC1A1*, and *MALAT1* in HK-2 (normal cells) and CAKI-1 (cell models of ccRCC). (e) Survival analysis of Riskscore in the TCGA datasets (KIRC). (f) Survival analysis of Riskscore in the independent verification set (GSE29609). ^*∗*^Compared with the HK-2 group, *P* < 0.05.

To study the prognosis of ccRCC based on metabolism, we established a Riskscore model according to the following steps. First, by analyzing the differential expression of metabolism-related genes between the above two categories, 667 candidate genes (Supplementary Table S2) were initially obtained; 408 genes (Supplementary Table S3) were left after univariate screening. Finally, 12 genes were obtained using the random forest algorithm (Figure 2(a)): *MDK*, *SLC1A1*, *SGCB*, *C4orf3*, *MALAT1*, *PILRB*, *IGHG1*, *FZD1*, *IFITM1*, *MUC20*, *KRT80*, and *SALL1*. The Riskscore model for these 12 genes was established using the multivariate Cox method. The Riskscore of each sample was calculated by the sum of multiplying the gene expression in the sample with their coefficient (the weight calculated by the Cox regression model). Then, we analyzed the correlation between Riskscores and the above 12 genes and ranked the samples according to the model score to create a heat map.  Figure 2(b) shows that these 12 genes have statistically significant Riskscores. The expression trends of six upregulated genes, *MDK*, *MALAT1*, *PILRB*, *IGHG1*, *IFITM1*, *KRT80*, and six downregulated genes, *SLC1A1*, *SGCB*, *C4orf3*, *FZD1*, *MUC20*, and *SALL1*, were consistent with the positive and negative coefficients in the model. We chose 4 genes, *SLC1A1*, *MALAT1*, *FZD1*, and *SALL1*, performed survival analysis, and compared the different expressions between HK-2 cells and CAKI-1 cells, to verify their importance. Patients with low *SLC1A1*, *FZD1*, or *SALL1* expression or high *MALAT1* expression have a poor prognosis (Figure 2(c) and Figure S3). Compared with HK-2 cells, *SLC1A1*, *FZD1*, and *SALL1* were low-expressed, while *MALAT1* was high-expressed in CAKI-1 cells (Figure 2(d)). The survival analysis of the Riskscore model in the TCGA dataset (Figure 2(e)) and independent validation set (Figure 2(f)) showed that the higher the Riskscore, the worse the prognosis of patients; the *P* values of <0.05 document statistical significance. This Riskscore model could be used to predict the prognosis of ccRCC.

### 3.3. Riskscore and Different Metabolic Patterns between ccRCC Samples

**Figure 3:**
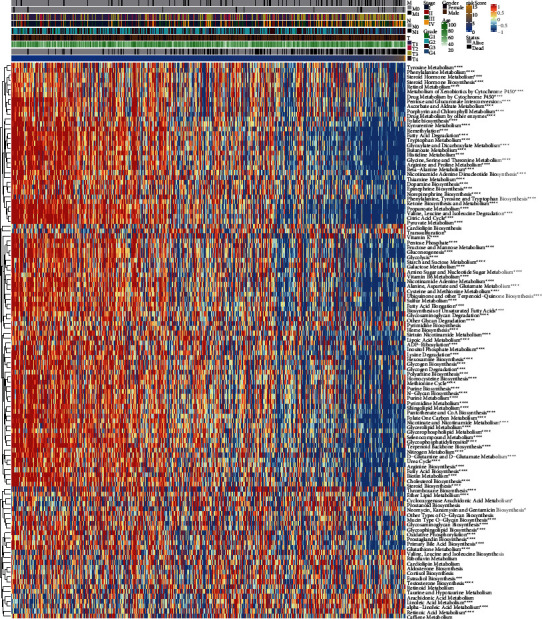
Relationship between Riskscore and metabolic patterns. The correlation analyses between Riskscore and the activity of 114 metabolic pathways.

The results of the correlation analysis between Riskscore and the activities of 114 metabolic pathways are shown in Figure 3. The Riskscore was negatively correlated with carbohydrate metabolism pathways such as glycolysis, gluconeogenesis, pyruvate metabolism, citric acid cycle, oxidative phosphorylation, pentose and glucuronate interconversions, and pentose phosphate and was negatively correlated with amino acid metabolism pathways such as glycine, serine and threonine metabolism, alanine, aspartate and glutamate metabolism, homocysteine biosynthesis, methionine cycle, cysteine and methionine metabolism, and kynurenine metabolism. The Riskscore was also negatively correlated with lipid metabolism pathways such as fatty acid degradation, glycerolipid metabolism, glycerophospholipid metabolism, steroid hormone metabolism, and steroid hormone biosynthesis, and negatively correlated with purine metabolism, pyrimidine metabolism, and purine biosynthesis. Riskscore was also negatively correlated with remethylation, vitamin K, retinol metabolism, and other pathways. But there were few pathways positively correlated with Riskscore, such as transsulfuration, thromboxane biosynthesis, linoleic acid metabolism, alpha-linoleic acid metabolism, cyclooxygenase arachidonic acid metabolism, and retinoic acid metabolism, etc. It can be seen that our Riskscore is related to most metabolic processes and is mainly negatively correlated, involving almost every category of metabolism.

### 3.4. Relationship between Immune Infiltration and Immune Regulatory Factors in ccRCC Riskscore

**Figure 4:**
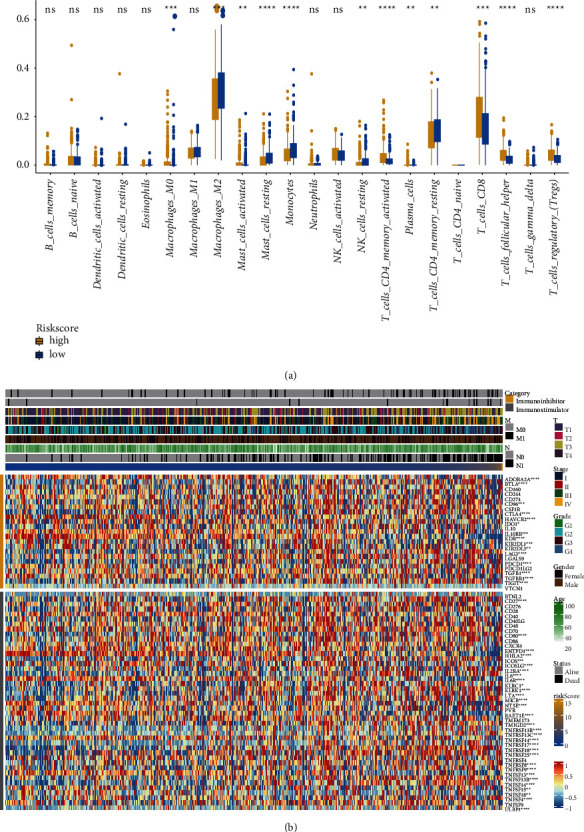
Relationship between Riskscore, immune infiltration, and immune checkpoints. (a) The high- and low-Riskscores of immune infiltrations. (b) Correlation between Riskscore and expression of immune regulatory factors.

To explore the relationship between Riskscore and immune infiltration, we divided the samples into two groups: high-Riskscore and low-Riskscore (Figure 4(a)). There were significant Riskscore differences in immune cells, including macrophages M0, macrophages M2, mast cells activated, mast cells resting, monocytes, NK cells resting, plasma cells, T cells CD4 memory activated, T cells CD4 memory resting, T cells CD8, T cells follicular helper, and T cells regulatory (Tregs). To explore the relationship between Riskscore and immune regulatory factors, we analyzed the correlation between Riskscore and immune inhibitors and immune stimulators and created a heat map (Figure 4(b)). The results show that our Riskscore model was related to immune inhibitors such as *ADORA2A*, *BTLA*, *CD96*, *CTLA4*, *HAVCR2*, *IDO1*, *IL10RB*, *KDR*, *KIR2DL1*, *KIR2DL3*, *LAG3*, *PDCD1*, *TGFB1*, *TGFBR1*, and *TIGIT*, and related to immune stimulators such as *CD27*, *CD80*, *ENTPD1*, *HHLA2*, *ICOS*, *ICOSLG*, *IL2RA*, *IL6*, *IL6R*, *KLRC1*, *KLRK1*, *LTA*, *MICB*, *NT5E*, *RAET1E*, *TMIGD2*, *TNFRSF13B*, *TNFRSF13C*, *TNFRSF14*, *TNFRSF17*, *TNFRSF18*, *TNFRSF25*, *TNFRSF8*, *TNFRSF9*, *TNFSF13*, *TNFSF13B*, *TNFSF14*, *TNFSF15*, *TNFSF18*, *TNFSF4*, and *ULBP1*. The Riskscore model we constructed was related to the immune infiltration and immune checkpoints.

### 3.5. Relationship between Riskscore and Clinical Features of ccRCC

**Figure 5:**
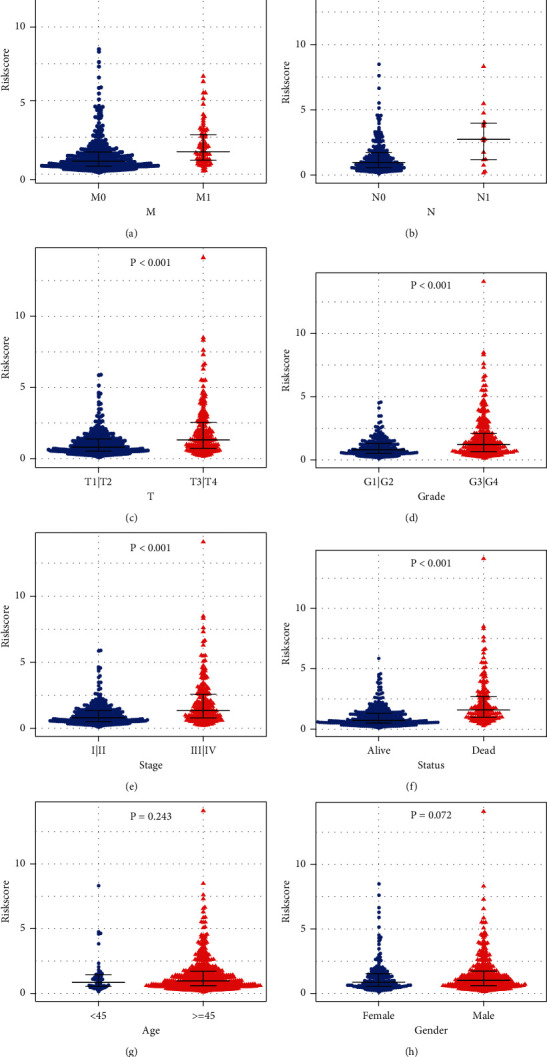
Difference in Riskscore model analyses for different clinical characteristics. (a) Different distributions of Riskscore in M0 and M1 stages. (b) Riskscore distribution among N0 and N1 stages. (c) Different distributions of Riskscore in T1 + T2 stage and T3 + T4 stages. (d) Riskscore distribution in G1 + G2 grade and G3 + G4 grade. (e) Riskscore distribution in stage I + stage II and stage III + stage IV. (f) Different distributions of Riskscore in alive and dead. (g) Different Riskscore distributions by age. (h) Different Riskscore by gender.

To analyze the relationship between different clinical characteristics and Riskscore model, we first classified the data according to clinical characteristics. The results of the data analysis showed no significant difference in Riskscore among patients of different ages or genders, but there was a significant difference in Riskscore among samples with different M stage, N stage, T stage, grade, stage, and status (Figure 5). Univariate and multivariate analyses of clinical features and Riskscores are shown in Table 2. Univariate analysis showed that the Riskscore was significantly correlated with all clinical features except gender, while multivariate analysis showed that the Riskscore was significantly correlated with age and M stage. The results above indicated that Riskscores based on these 12 metabolism-related genes were a feasible prognostic factor in different populations.

## 4. Discussion

Metabolic reprogramming usually occurs in tumors. Compared with other cancers, studies tend to regard ccRCC as a metabolic disease [31, 32]. Metabonomics experiments have confirmed that there are significant changes in metabolic patterns in ccRCC, such as the rapid destruction of metabolic pathways of energy, amino acids, creatinine, and uric acid [33]. Many studies have evaluated the prognosis and diagnosis of ccRCC from the perspective of metabolic patterns [2] and treatment of ccRCC by reversing the abnormal metabolic pattern [34]. Our study has established a metabolic model to assess the prognosis of ccRCC and identified several genes related to the disease that were not considered previously.

Among the genes we selected to construct the Riskscore model, *SLC1A1* [35], *FZD1* [36], and *SALL1* [37] were downregulated in ccRCC, while *MALAT1* [38] was upregulated; there are specific clinical trials to verify their relationship with ccRCC. For example, *MALAT1* is a lncRNA that acts as a marker in various cancers [39], and miR-182-5p can reduce the proliferation of ccRCC by binding with *MALAT1* [38]. *C4orf3* [40] and *MDK* [41] were also used as markers to evaluate ccRCC. Research on the remaining genes in ccRCC is rare, although these genes play important roles in other cancers.

It seems that cancers prefer glycolysis that does not require oxygen consumption and does not involve pyruvate metabolism [42], and this phenomenon was confirmed by isotope experiments in ccRCC [43]. Oxidative phosphorylation is not high in ccRCC [4], and the TCA cycle in ccRCC is also reduced, differing from the metabolic pattern of the human brain and lung tumors [43]. The inhibition of gluconeogenesis and increased glycolysis are common in ccRCC [44]. In more than 600 cases of ccRCC, the level of fructose 1,6-bisphosphatase 1 (FBP1), a gluconeogenic enzyme, was reduced and was related to the poor prognosis of this disease [45]. In addition to the glycolysis pathway, the negative trends of our Riskscore model were consistent with those reported above for carbohydrate metabolism. Serine and threonine that often appear at protein kinase phosphorylation sites have hydroxyl groups in their structures. Although serine and glycine are nonessential amino acids, many cells still rely on exogenous serine to achieve optimal growth. Some studies have attempted to reduce the intake of amino acids in the diet to alleviate cancer [46]. The tryptophan level in ccRCC was decreased and its metabolism was strikingly linked to the kynurenine pathway [32]. Kynurenine metastasis of tumor cells can upregulate programmed cell death-1 (PD-1) in T cells [47]. The significant increase in glutathione (GSH), a reactive oxygen species (ROS) scavenger, has been identified as a marker of RCC. Demand for cysteine also increases in ccRCC. Cysteine is synthesized via transsulfuration. The flux of the pentose phosphate pathway in ccRCC increases [45]. In addition to the pentose phosphate pathway and kynurenine pathway, the negative trends of our Riskscore model were consistent with the above reports on amino acid metabolism, and the positive trends of our Riskscore model and transsulfuration were also consistent with these reports. Lipid accumulation in ccRCC causes hypertrophy caused by impaired lipid metabolism [42, 48], mainly due to inhibition of *β*-oxidation [13, 48] and impairment of fatty acid degradation [48]. The negative trends of our Riskscore model were consistent with these reports on lipid metabolism. The expression of NT5E and ENTPD1, factors related to purine and pyrimidine metabolism, increases in ccRCC [49]. The negative trends of our Riskscore model were opposite to the above-reported nucleotide metabolism.

The effects of NK cells and neutrophils were related to the two studies clusters, but there were no significant differences between high and low Riskscores. M1 macrophages can inhibit tumors, whereas polarized M2 macrophages can promote tumors [50]. Tumor cells and macrophages produce complement C1q to promote tumor growth [51]. Consistent with the Riskscores trends, M0 macrophages and T follicle helper cells in high-risk patients were higher than those in low-risk patients [52]. However, in our study, cells with a low-Riskscore had more M2 macrophages in ccRCC. CD138^+^ plasma cells may secrete antibodies or act as Breg cells and promote tumor growth [53]. Consistent with this trend, our study found that the higher the Riskscore, the higher the number of plasma cells present. Activated CD4^+^ memory T lymphocytes can target antigenic tumor cells, inhibit tumor growth, and play an active regulatory role in anti-tumor immunity [54]. However, in our study, the higher the Riskscore, the more of these two cell types were involved. There may be cooperation between immune cells that increases the complexity, and even the immune cells may be further divided into more subtypes, so the observation may not be simply related to the number of cells of a given type. The overall survival and progression-free survival of patients with ccRCC and Hodgkin's lymphoma with severe CD8^+^ T cell infiltration were significantly shorter. However, in some ccRCC patients with a “normal” immune environment, oligoclonal CD8 T cells express perforin. A high density of CD8^+^ T cells is associated with a good prognosis in this subgroup [55]. The trends of our Riskscore model were consistent with those reported above.

The growth and progression of cancer are associated with immunosuppression [56]. Studies have found that immune inhibitors such as *LAG3* [27, 52], *BTLA* [27], *PD-1* (*PDCD1*) [52], and *CTLA-4* [52], or immunostimulators like *TNFSF13B* [57] play important roles in ccRCC. Our Riskscore was related to the immune checkpoints, which provides a new way to explore the mechanism of ccRCC.

The pattern of metabolism in ccRCC cells influences immune cells. For example, sulfatide, a product of ether lipid metabolism, accumulates in ccRCC, which could combine with platelets and evade cytotoxicity mediated by natural killer cells and immune surveillance [58]. In our study, we found that the effect of CD8 T cells was significantly higher in the high-Riskscore group (Figure 4(a)). Effector T cells require a high rate of glucose metabolism, while cancer cells inhibit T cells through using up nutrition and producing harmful components such as lactic acid. Although many CD8^+^ T cells are involved in ccRCC, they cannot take glucose or glycolysis effectively [59]. Glutamine addiction is a characteristic of ccRCC; running out of glutamine in the tumor microenvironment leads to the secretion of IL-23 by macrophages, activating Treg responses, and thereby suppressing the anti-tumor toxicity of T cells [60].

Different types of cells are distributed in different parts of many tumors, including ccRCC. The heterogeneity of cell type may directly lead to the heterogeneity of metabolism in different parts of the same tumor; for example, the clear cells in ccRCC should respond to angiogenesis and glycolysis inhibitors, while eosinophilic components in ccRCC may benefit from mTOR or glutaminase inhibition [61]. Although the Riskscore model was based on the expression differences of metabolic genes, and the relationship between the Riskscore model and the prognosis was roughly in line with our prediction after verification, the relationship between our model and metabolic pathways was not the same as the actual changes in ccRCC metabolic patterns, indicating the metabolic complexity of different stages of ccRCC. Our study suggests that these genes might be important, but further studies are needed to clarify and validate the detailed mechanism behind their indicated significance.

## 5. Conclusion

In this study, we constructed a Riskscore model with 12 metabolism-related genes. The higher the score, the worse the prognosis. The Riskscore is closely related to metabolism, immune infiltration, and immune checkpoints, which can be used as one of the potential prognostic criteria of ccRCC.

## Figures and Tables

**Table 1:** Primers used in this study.

Primers	Sequences	Product length
SLC1A1-F	5′- TGAAGCCTCCCAGCGATCCAG -3′	142 bp
SLC1A1-R	5′- ATCAAGCCCAGGACGTTTATGCC -3′	
MALAT1-F	5′- ACTGTTCTGATCCCGCTGCT -3′	136 bp
MALAT1-R	5′- CCTCAACACTCAGCCTTTATCACT -3′	
FZD1-F	5′- ACCAACAGCAAACAAGGGGA -3′	163 bp
FZD1-R	5′- GGAGCCTGCGAAAGAGAGTT -3′	
SALL1-F	5′- AAACGGACGGGGAAAGTGTC -3′	180 bp
SALL1-R	5′- CAAAGAACTCGGCACAGCAC -3′	
*β*-Actin-F	5′- ACCCTGAAGTACCCCATCGAG -3′	224 bp
*β*-Actin-R	5′- AGCACAGCCTGGATAGCAAC -3′	

**Table 2:** Univariate and multivariate analyses of Riskscore and clinicopathological features with overall survival in TCGA KIRC cohort.

Characteristics	Univariate analysis HR (95% CI)	*P* value	Multivariate analysis HR (95% CI)	*P* value
Riskscore	1.317 (1.249–1.389)	<0.001	1.216 (1.118–1.323)	<0.001
Age	1.030 (1.017–1.043)	<0.001	1.031 (1.012–1.052)	0.002
Gender	0.949 (0.696–1.294)	0.742	1.314 (0.839–2.059)	0.233
Grade	2.578 (1.835–3.624)	<0.001	1.401 (0.843–2.328)	0.193
Stage	3.804 (2.772–5.221)	<0.001	1.266 (0.491–3.266)	0.625
T stage	3.119 (2.303–4.225)	<0.001	1.184 (0.515–2.722)	0.691
N stage	3.394 (1.754–6.567)	<0.001	1.582 (0.762–3.285)	0.218
M stage	4.283 (3.131–5.860)	<0.001	3.109 (1.823–5.303)	<0.001

## Data Availability

All the data used in this study have been listed in the main text of the article. TCGA KIRC datasets were downloaded from UCSC Xena (https://xenabrowser.net/) as the training dataset for ccRCC, while GSE29609 was downloaded from the GEO database (https://www.ncbi.nlm.nih.gov/geo/) as the independent validation dataset.

## Supplementary Materials

Supplementary Materials Supplementary Table S1: clinicopathologic parameters of TCGA KIRC dataset. Supplementary Table S2: DEG to clusters. Supplementary Table S3: univar result after limma final. Supplementary Figure S1: flowchart of data collection and analysis in this study. Supplementary Figure S2: the relationship between the cophenetic coefficient and the number of clusters. Supplementary Figure S3: survival analysis of *MDK*, *SGCB*, *C4orf3*, *PILRB*, *IGHG1*, *IFITM1*, *MUC20*, and *KRT80*.
