# Supplementary material for: Identification of Prognostic Metabolism-Related Genes in Clear Cell Renal Cell Carcinoma
Source: J Oncol. 2021 Sep 27;2021:2042114. doi: 10.1155/2021/2042114 (PMC8490028; doi:10.1155/2021/2042114)
Supplement: Supplementary Materials — Supplementary Table S1: clinicopathologic parameters of TCGA KIRC dataset. Supplementary Table S2: DEG to clusters. Supplementary Table S3: univar result after limma final. Supplementary Figure S1: flowchart of data collection and analysis in this study. Supplementary Figure S2: the relationship between the cophenetic coefficient and the number of clusters. Supplementary Figure S3: survival analysis of MDK, SGCB, C4orf3, PILRB, IGHG1, IFITM1, MUC20, and KRT80. [file 2042114.f1.zip › 2042114.f1/Supplementary Table S1.docx]

Supplemental Table S1. Clinicopathologic parameters of TCGA KIRC dataset.

| Features |  | Case (n=529) | High Riskscore (n=316) | Low Riskscore (n=213) | *P* value |
| --- | --- | --- | --- | --- | --- |
| Gender |  |  |  |  | 0.0728 |
|  | Male | 345 | 218 | 127 |  |
|  | Female | 184 | 98 | 86 |  |
| Age |  |  |  |  | 0.9597 |
|  | <65 | 331 | 198 | 133 |  |
|  | ≥65 | 198 | 118 | 80 |  |
| Grade |  |  |  |  | <0.0001 |
|  | N/A | 8 | 3 | 5 |  |
|  | G1 | 13 | 5 | 8 |  |
|  | G2 | 228 | 118 | 110 |  |
|  | G3 | 206 | 126 | 80 |  |
|  | G4 | 74 | 64 | 10 |  |
| Stage |  |  |  |  | <0.0001 |
|  | N/A | 3 | 2 | 1 |  |
|  | I | 265 | 129 | 136 |  |
|  | II | 57 | 34 | 23 |  |
|  | III | 123 | 85 | 38 |  |
|  | IV | 81 | 66 | 15 |  |
| Status |  |  |  |  | <0.0001 |
|  | Alive | 356 | 165 | 191 |  |
|  | Dead | 173 | 151 | 22 |  |
