# Supplementary material for: Identification of Prognostic Metabolism-Related Genes in Clear Cell Renal Cell Carcinoma
Source: J Oncol. 2021 Sep 27;2021:2042114. doi: 10.1155/2021/2042114 (PMC8490028; doi:10.1155/2021/2042114)
Supplement: Supplementary Materials — Supplementary Table S1: clinicopathologic parameters of TCGA KIRC dataset. Supplementary Table S2: DEG to clusters. Supplementary Table S3: univar result after limma final. Supplementary Figure S1: flowchart of data collection and analysis in this study. Supplementary Figure S2: the relationship between the cophenetic coefficient and the number of clusters. Supplementary Figure S3: survival analysis of MDK, SGCB, C4orf3, PILRB, IGHG1, IFITM1, MUC20, and KRT80. [file 2042114.f1.zip › 2042114.f1/Supplementary Table S2.docx]

Supplemental Table S2. DEG to clusters.

| symbol | logFC | AveExpr | t | P.Value | adj.P.Val | B |
| --- | --- | --- | --- | --- | --- | --- |
| TPI1 | -0.77043 | 10.16211 | -16.5634 | 6.20E-50 | 1.13E-45 | 102.6132 |
| DDOST | -0.62634 | 7.277165 | -15.5691 | 2.95E-45 | 2.68E-41 | 91.98488 |
| PKM | -0.66204 | 10.16126 | -14.3888 | 7.60E-40 | 4.32E-36 | 79.68921 |
| PDIA6 | -0.82072 | 7.309885 | -13.9166 | 9.89E-38 | 3.60E-34 | 74.88487 |
| P4HA1 | -0.98343 | 6.010547 | -13.6961 | 9.37E-37 | 2.85E-33 | 72.66662 |
| PGAM1 | -0.79388 | 8.270658 | -13.4935 | 7.29E-36 | 1.66E-32 | 70.64286 |
| LRRC42 | -0.6693 | 4.022258 | -13.3898 | 2.07E-35 | 4.19E-32 | 69.6129 |
| CLIC1 | -0.58961 | 8.674336 | -13.3557 | 2.92E-35 | 5.31E-32 | 69.2754 |
| PGK1 | -0.96278 | 9.451029 | -13.2421 | 9.10E-35 | 1.51E-31 | 68.15277 |
| LDHA | -0.91416 | 10.51068 | -13.0566 | 5.78E-34 | 6.58E-31 | 66.3298 |
| ENO1 | -0.8775 | 10.77087 | -13.0223 | 8.13E-34 | 7.79E-31 | 65.99383 |
| SLC16A3 | -1.11052 | 7.262322 | -12.8338 | 5.23E-33 | 4.54E-30 | 64.15728 |
| TMEM9 | -0.59798 | 6.495417 | -12.7485 | 1.21E-32 | 8.81E-30 | 63.33099 |
| TAGLN2 | -0.66251 | 8.586258 | -12.7176 | 1.64E-32 | 1.10E-29 | 63.03232 |
| EIF4A1 | -0.5883 | 8.656005 | -12.6724 | 2.55E-32 | 1.60E-29 | 62.59634 |
| LMAN2 | -0.59187 | 7.558919 | -12.6664 | 2.70E-32 | 1.64E-29 | 62.53894 |
| PRDX4 | -0.63125 | 7.020975 | -12.5955 | 5.40E-32 | 2.96E-29 | 61.85636 |
| TNFRSF1A | -0.63077 | 6.794342 | -12.4874 | 1.54E-31 | 6.86E-29 | 60.81985 |
| TCTN3 | -0.58834 | 5.082442 | -12.3412 | 6.35E-31 | 2.51E-28 | 59.42615 |
| PHC2 | -0.6036 | 6.496574 | -12.1867 | 2.80E-30 | 9.45E-28 | 57.96288 |
| HK1 | -0.67467 | 5.504099 | -12.1107 | 5.79E-30 | 1.76E-27 | 57.24651 |
| SLC2A1 | -1.20489 | 7.223287 | -12.096 | 6.67E-30 | 1.96E-27 | 57.10802 |
| YWHAQ | -0.677 | 7.311709 | -12.0774 | 7.96E-30 | 2.26E-27 | 56.93359 |
| VKORC1 | -0.60441 | 7.425241 | -11.9692 | 2.23E-29 | 5.71E-27 | 55.91998 |
| TPM3 | -0.60987 | 7.638454 | -11.8725 | 5.56E-29 | 1.28E-26 | 55.01838 |
| PGM1 | -0.718 | 5.833951 | -11.8541 | 6.61E-29 | 1.50E-26 | 54.84757 |
| HDGF | -0.63584 | 7.296499 | -11.843 | 7.34E-29 | 1.65E-26 | 54.74441 |
| S100A10 | -0.62585 | 9.535168 | -11.8117 | 9.85E-29 | 2.16E-26 | 54.45414 |
| ANXA2 | -0.68288 | 8.986133 | -11.7833 | 1.29E-28 | 2.66E-26 | 54.19061 |
| MSH6 | -0.67206 | 3.867909 | -11.6742 | 3.58E-28 | 6.44E-26 | 53.18362 |
| JPT2 | -0.67499 | 5.672547 | -11.5999 | 7.15E-28 | 1.20E-25 | 52.50053 |
| RPS12 | -0.60126 | 10.21195 | -11.5859 | 8.15E-28 | 1.34E-25 | 52.37167 |
| LAPTM4A | -0.5975 | 8.25947 | -11.5674 | 9.68E-28 | 1.55E-25 | 52.2024 |
| DDX50 | -0.6057 | 4.396241 | -11.5227 | 1.47E-27 | 2.25E-25 | 51.7938 |
| CKS1B | -0.65997 | 5.421453 | -11.5085 | 1.67E-27 | 2.50E-25 | 51.66418 |
| ASCC1 | -0.62986 | 4.498992 | -11.4535 | 2.78E-27 | 3.96E-25 | 51.16229 |
| PLOD1 | -0.80548 | 6.800355 | -11.4182 | 3.85E-27 | 5.16E-25 | 50.84125 |
| TMED2 | -0.60757 | 6.944002 | -11.3974 | 4.67E-27 | 5.99E-25 | 50.65199 |
| RRM1 | -0.59024 | 4.312744 | -11.2808 | 1.36E-26 | 1.59E-24 | 49.59644 |
| TUBB | -0.58592 | 8.04756 | -11.2796 | 1.38E-26 | 1.60E-24 | 49.58603 |
| SERPINB8 | -0.61577 | 2.861235 | -11.21 | 2.61E-26 | 2.91E-24 | 48.95894 |
| PRPS1 | -0.59048 | 4.664673 | -11.2002 | 2.85E-26 | 3.11E-24 | 48.8715 |
| PANX1 | -0.62263 | 3.208456 | -11.1102 | 6.46E-26 | 6.50E-24 | 48.06421 |
| INSIG2 | -0.67722 | 5.165835 | -11.0846 | 8.15E-26 | 7.98E-24 | 47.83579 |
| TUBA1B | -0.69927 | 9.140906 | -11.07 | 9.31E-26 | 8.98E-24 | 47.70529 |
| ACLY | -0.90722 | 6.778477 | -11.0657 | 9.67E-26 | 9.27E-24 | 47.66743 |
| SNX7 | -0.75926 | 4.740797 | -11.0574 | 1.04E-25 | 9.93E-24 | 47.59336 |
| H3F3B | -0.61151 | 8.90506 | -10.9616 | 2.48E-25 | 2.20E-23 | 46.74143 |
| S100A16 | -0.68608 | 6.914263 | -10.9135 | 3.82E-25 | 3.29E-23 | 46.31606 |
| MPZL1 | -0.73478 | 5.800782 | -10.9065 | 4.06E-25 | 3.47E-23 | 46.25423 |
| OSMR | -0.89583 | 5.189725 | -10.8842 | 4.96E-25 | 4.15E-23 | 46.05697 |
| TXNDC5 | -0.71599 | 6.547173 | -10.8545 | 6.48E-25 | 5.20E-23 | 45.79497 |
| MCM5 | -0.58697 | 4.615903 | -10.8461 | 6.98E-25 | 5.55E-23 | 45.72127 |
| SERBP1 | -0.62339 | 6.677097 | -10.8442 | 7.10E-25 | 5.62E-23 | 45.70451 |
| AK4 | -1.00744 | 5.413318 | -10.7728 | 1.34E-24 | 9.94E-23 | 45.07755 |
| DPCD | -0.65981 | 5.51053 | -10.7285 | 1.99E-24 | 1.42E-22 | 44.68903 |
| RPA1 | -0.60304 | 5.100801 | -10.6927 | 2.74E-24 | 1.91E-22 | 44.37694 |
| PPT1 | -0.62221 | 6.556175 | -10.6871 | 2.88E-24 | 2.00E-22 | 44.32739 |
| C5orf15 | -0.71664 | 6.408717 | -10.6782 | 3.11E-24 | 2.13E-22 | 44.25016 |
| PLIN3 | -0.60962 | 5.003734 | -10.6694 | 3.36E-24 | 2.28E-22 | 44.17354 |
| CDC42EP4 | -0.64232 | 4.38844 | -10.5564 | 9.12E-24 | 5.69E-22 | 43.19189 |
| RCN1 | -0.58627 | 6.364667 | -10.5478 | 9.83E-24 | 6.07E-22 | 43.1179 |
| WDR54 | -0.7468 | 5.122633 | -10.4728 | 1.90E-23 | 1.12E-21 | 42.47081 |
| MCMBP | -0.59325 | 4.453821 | -10.452 | 2.28E-23 | 1.33E-21 | 42.29167 |
| C12orf57 | -0.59231 | 7.749564 | -10.4409 | 2.51E-23 | 1.44E-21 | 42.19615 |
| ERGIC1 | -0.64505 | 7.349055 | -10.412 | 3.23E-23 | 1.80E-21 | 41.94792 |
| GDI2 | -0.60709 | 7.041242 | -10.4102 | 3.28E-23 | 1.82E-21 | 41.93289 |
| ISG20L2 | -0.60794 | 4.041908 | -10.3811 | 4.22E-23 | 2.30E-21 | 41.68348 |
| C4orf3 | -0.60839 | 6.638619 | -10.3654 | 4.84E-23 | 2.57E-21 | 41.54855 |
| RNF145 | -0.59692 | 5.713164 | -10.2984 | 8.66E-23 | 4.37E-21 | 40.97753 |
| PFKFB4 | -0.74393 | 3.375014 | -10.2856 | 9.68E-23 | 4.83E-21 | 40.86794 |
| SHC1 | -0.59585 | 6.297643 | -10.2766 | 1.05E-22 | 5.17E-21 | 40.79193 |
| MPI | -0.61913 | 5.921246 | -10.2745 | 1.06E-22 | 5.24E-21 | 40.77412 |
| SAP30 | -0.69609 | 4.401892 | -10.2357 | 1.49E-22 | 7.25E-21 | 40.4441 |
| DHRS13 | -0.61225 | 2.858159 | -10.1545 | 2.99E-22 | 1.38E-20 | 39.75759 |
| PIGS | -0.67711 | 5.291562 | -10.0873 | 5.32E-22 | 2.34E-20 | 39.19234 |
| TSPAN4 | -0.63336 | 6.563366 | -10.0327 | 8.47E-22 | 3.60E-20 | 38.73412 |
| SSR1 | -0.65038 | 5.740141 | -10.0287 | 8.76E-22 | 3.71E-20 | 38.70127 |
| BNIP3 | -0.83586 | 6.972784 | -10.0267 | 8.91E-22 | 3.76E-20 | 38.68404 |
| PDK1 | -0.7191 | 4.134541 | -9.99821 | 1.14E-21 | 4.73E-20 | 38.44614 |
| CCND3 | -0.61679 | 5.527249 | -9.99318 | 1.18E-21 | 4.90E-20 | 38.40417 |
| VIM | -0.81333 | 11.15609 | -9.98349 | 1.29E-21 | 5.30E-20 | 38.32326 |
| PLSCR1 | -0.66059 | 6.11517 | -9.97931 | 1.33E-21 | 5.47E-20 | 38.2884 |
| CFAP36 | -0.59665 | 5.992385 | -9.96096 | 1.56E-21 | 6.34E-20 | 38.13551 |
| HK2 | -0.94443 | 4.170427 | -9.96034 | 1.57E-21 | 6.36E-20 | 38.13042 |
| FNDC3B | -0.63096 | 4.849414 | -9.93744 | 1.90E-21 | 7.62E-20 | 37.93988 |
| BTG1 | -0.67111 | 6.606068 | -9.91672 | 2.26E-21 | 8.92E-20 | 37.76771 |
| CXCL16 | -0.68924 | 5.807463 | -9.85502 | 3.81E-21 | 1.42E-19 | 37.25668 |
| ERLIN1 | -0.67629 | 3.996105 | -9.84723 | 4.06E-21 | 1.50E-19 | 37.1923 |
| PDIA4 | -0.63444 | 6.365155 | -9.84193 | 4.25E-21 | 1.57E-19 | 37.14847 |
| G3BP1 | -0.61575 | 5.49284 | -9.82869 | 4.75E-21 | 1.74E-19 | 37.03917 |
| NNMT | -1.35959 | 10.0401 | -9.82253 | 5.00E-21 | 1.82E-19 | 36.98836 |
| DDIT4 | -0.89077 | 8.11584 | -9.80215 | 5.93E-21 | 2.13E-19 | 36.82048 |
| ORAI2 | -0.59538 | 3.802483 | -9.79166 | 6.48E-21 | 2.30E-19 | 36.73405 |
| TUBA1A | -0.66575 | 7.206553 | -9.78866 | 6.64E-21 | 2.35E-19 | 36.7094 |
| IFT57 | -0.61331 | 4.730535 | -9.78747 | 6.71E-21 | 2.36E-19 | 36.69959 |
| SKP1 | -0.60136 | 8.740009 | -9.77603 | 7.38E-21 | 2.57E-19 | 36.60549 |
| RIMKLA | -0.76253 | 2.230976 | -9.77601 | 7.38E-21 | 2.57E-19 | 36.60533 |
| FAS | -0.71505 | 4.299034 | -9.75973 | 8.46E-21 | 2.90E-19 | 36.47161 |
| PAM | -0.62433 | 7.484484 | -9.72902 | 1.09E-20 | 3.66E-19 | 36.21971 |
| TOMM20 | -0.60704 | 6.676112 | -9.72093 | 1.17E-20 | 3.90E-19 | 36.15347 |
| HSPA13 | -0.60186 | 4.070389 | -9.64379 | 2.22E-20 | 6.99E-19 | 35.52359 |
| SSPN | -0.80135 | 3.913259 | -9.63392 | 2.41E-20 | 7.55E-19 | 35.4433 |
| CREG1 | -0.62234 | 6.700115 | -9.61515 | 2.81E-20 | 8.74E-19 | 35.29065 |
| ASPH | -0.6885 | 5.985825 | -9.60453 | 3.07E-20 | 9.50E-19 | 35.20439 |
| MCFD2 | -0.63978 | 5.791225 | -9.596 | 3.30E-20 | 1.02E-18 | 35.13516 |
| NRAS | -0.59785 | 4.47995 | -9.56073 | 4.41E-20 | 1.33E-18 | 34.84932 |
| GALNT1 | -0.59264 | 4.651551 | -9.5538 | 4.67E-20 | 1.39E-18 | 34.79326 |
| ODC1 | -0.68561 | 5.695899 | -9.53513 | 5.44E-20 | 1.60E-18 | 34.64227 |
| SDC3 | -0.67077 | 4.568591 | -9.50441 | 7.00E-20 | 2.02E-18 | 34.39441 |
| MSN | -0.6146 | 7.856765 | -9.49622 | 7.49E-20 | 2.13E-18 | 34.32841 |
| TUBA1C | -0.68581 | 7.4239 | -9.48411 | 8.27E-20 | 2.33E-18 | 34.2309 |
| CANX | -0.68454 | 8.449654 | -9.47819 | 8.68E-20 | 2.44E-18 | 34.18332 |
| RALB | -0.6059 | 6.047763 | -9.42401 | 1.35E-19 | 3.69E-18 | 33.74833 |
| ERAP1 | -0.69426 | 4.755832 | -9.41532 | 1.45E-19 | 3.93E-18 | 33.67876 |
| PFKP | -0.79032 | 7.767295 | -9.40077 | 1.63E-19 | 4.39E-18 | 33.56231 |
| ADAM17 | -0.59274 | 3.301908 | -9.3803 | 1.93E-19 | 5.09E-18 | 33.39869 |
| NOTCH2 | -0.71648 | 3.869451 | -9.35439 | 2.38E-19 | 6.20E-18 | 33.19197 |
| WWTR1 | -0.76374 | 6.095618 | -9.35185 | 2.43E-19 | 6.30E-18 | 33.17177 |
| MTDH | -0.74006 | 5.069106 | -9.34905 | 2.49E-19 | 6.42E-18 | 33.14945 |
| TMED10 | -0.60368 | 7.019917 | -9.33854 | 2.71E-19 | 6.92E-18 | 33.06578 |
| TNFAIP8 | -0.64668 | 4.741105 | -9.33847 | 2.71E-19 | 6.92E-18 | 33.0652 |
| BNIP3L | -0.7604 | 6.787482 | -9.33284 | 2.84E-19 | 7.22E-18 | 33.02043 |
| SLC16A1 | -0.71282 | 4.731109 | -9.32196 | 3.10E-19 | 7.77E-18 | 32.93386 |
| LRP1 | -0.67782 | 5.451788 | -9.31663 | 3.23E-19 | 8.06E-18 | 32.89148 |
| F11R | -0.61364 | 5.165917 | -9.31012 | 3.41E-19 | 8.45E-18 | 32.8398 |
| PFKFB3 | -0.80006 | 5.785662 | -9.29918 | 3.73E-19 | 9.12E-18 | 32.75291 |
| RNF19B | -0.67114 | 4.190961 | -9.29773 | 3.77E-19 | 9.18E-18 | 32.74141 |
| PTPN14 | -0.64433 | 3.045741 | -9.27459 | 4.54E-19 | 1.08E-17 | 32.55799 |
| YIPF5 | -0.66429 | 4.930639 | -9.25809 | 5.19E-19 | 1.22E-17 | 32.42738 |
| CAND1 | -0.61603 | 4.903893 | -9.25792 | 5.20E-19 | 1.22E-17 | 32.42604 |
| NPM1 | -0.61684 | 10.35316 | -9.23554 | 6.22E-19 | 1.43E-17 | 32.24911 |
| CD300A | -0.75259 | 3.937067 | -9.22941 | 6.54E-19 | 1.49E-17 | 32.20076 |
| NEK6 | -0.8306 | 5.626527 | -9.22796 | 6.61E-19 | 1.51E-17 | 32.1893 |
| BZW1 | -0.70335 | 6.048579 | -9.2124 | 7.49E-19 | 1.69E-17 | 32.06659 |
| LEPROT | -0.60616 | 5.790059 | -9.20681 | 7.84E-19 | 1.76E-17 | 32.02248 |
| B4GALT1 | -0.6059 | 5.634199 | -9.19914 | 8.34E-19 | 1.86E-17 | 31.96208 |
| GPX8 | -0.72362 | 4.18527 | -9.17824 | 9.85E-19 | 2.17E-17 | 31.79765 |
| BST2 | -0.79369 | 7.35069 | -9.17549 | 1.01E-18 | 2.20E-17 | 31.77607 |
| MYOF | -0.68635 | 5.40708 | -9.15012 | 1.23E-18 | 2.64E-17 | 31.57686 |
| GALNT18 | -0.73991 | 4.507006 | -9.14745 | 1.26E-18 | 2.68E-17 | 31.55591 |
| NDRG1 | -0.82329 | 9.849233 | -9.14699 | 1.27E-18 | 2.69E-17 | 31.55228 |
| RECQL | -0.65554 | 4.153646 | -9.14228 | 1.31E-18 | 2.78E-17 | 31.51539 |
| PMP22 | -0.72649 | 5.719314 | -9.13056 | 1.44E-18 | 3.03E-17 | 31.42348 |
| ITGB1 | -0.62447 | 8.033952 | -9.12021 | 1.57E-18 | 3.26E-17 | 31.34252 |
| ANO6 | -0.64398 | 5.338382 | -9.1176 | 1.60E-18 | 3.31E-17 | 31.32209 |
| TMEM45A | -1.23539 | 4.591489 | -9.11646 | 1.61E-18 | 3.33E-17 | 31.31318 |
| PAPSS2 | -0.74261 | 3.64911 | -9.09056 | 1.98E-18 | 4.04E-17 | 31.11076 |
| TMEM183B | -0.59196 | 2.266216 | -9.07032 | 2.33E-18 | 4.68E-17 | 30.95285 |
| WDR3 | -0.67748 | 2.812173 | -9.04156 | 2.93E-18 | 5.80E-17 | 30.72893 |
| CASP1 | -0.63236 | 4.94216 | -9.04088 | 2.94E-18 | 5.82E-17 | 30.72362 |
| EVA1A | -0.71457 | 4.507959 | -9.01436 | 3.63E-18 | 7.05E-17 | 30.5177 |
| CHSY1 | -0.67168 | 4.497299 | -9.00241 | 3.99E-18 | 7.69E-17 | 30.42496 |
| PLOD2 | -0.83038 | 6.479609 | -8.97761 | 4.85E-18 | 9.18E-17 | 30.23291 |
| TGOLN2 | -0.62445 | 6.311759 | -8.94709 | 6.17E-18 | 1.14E-16 | 29.99712 |
| FPR1 | -0.88582 | 2.920115 | -8.94641 | 6.20E-18 | 1.15E-16 | 29.99184 |
| PYGL | -0.71204 | 5.032285 | -8.93259 | 6.92E-18 | 1.27E-16 | 29.88526 |
| CALU | -0.62838 | 5.99854 | -8.92529 | 7.32E-18 | 1.33E-16 | 29.82907 |
| ESYT2 | -0.58653 | 5.289354 | -8.91503 | 7.94E-18 | 1.44E-16 | 29.75003 |
| LRP10 | -0.66021 | 5.524449 | -8.91359 | 8.03E-18 | 1.45E-16 | 29.73895 |
| ANTXR2 | -0.73496 | 4.469616 | -8.88057 | 1.04E-17 | 1.85E-16 | 29.48517 |
| APP | -0.61149 | 9.000821 | -8.86434 | 1.18E-17 | 2.07E-16 | 29.36076 |
| OAS3 | -0.64287 | 3.586221 | -8.85003 | 1.32E-17 | 2.30E-16 | 29.25112 |
| MARVELD1 | -0.65772 | 4.024007 | -8.84638 | 1.36E-17 | 2.36E-16 | 29.2232 |
| KCTD17 | -0.58867 | 3.283354 | -8.81924 | 1.68E-17 | 2.86E-16 | 29.01573 |
| NT5E | -0.75351 | 3.622388 | -8.81582 | 1.72E-17 | 2.94E-16 | 28.98964 |
| CMTM3 | -0.61965 | 4.951827 | -8.77915 | 2.29E-17 | 3.81E-16 | 28.71014 |
| TRIP12 | -0.6103 | 5.324223 | -8.77675 | 2.33E-17 | 3.88E-16 | 28.69185 |
| PIK3CB | -0.59214 | 4.520819 | -8.7712 | 2.44E-17 | 4.05E-16 | 28.64965 |
| FTO | -0.64886 | 5.661029 | -8.76918 | 2.48E-17 | 4.11E-16 | 28.63431 |
| SLC33A1 | -0.62201 | 4.316541 | -8.76509 | 2.55E-17 | 4.23E-16 | 28.6032 |
| SECTM1 | -0.80664 | 3.662396 | -8.75821 | 2.69E-17 | 4.42E-16 | 28.55091 |
| EXT1 | -0.5943 | 4.327362 | -8.75354 | 2.79E-17 | 4.57E-16 | 28.51548 |
| THEMIS2 | -0.66772 | 4.429314 | -8.73424 | 3.24E-17 | 5.24E-16 | 28.36908 |
| TGM2 | -0.87262 | 6.72112 | -8.7259 | 3.46E-17 | 5.54E-16 | 28.30583 |
| P4HA2 | -0.65939 | 6.00815 | -8.7027 | 4.14E-17 | 6.50E-16 | 28.13028 |
| DDX58 | -0.5855 | 3.461398 | -8.69517 | 4.39E-17 | 6.85E-16 | 28.07336 |
| TNFSF10 | -0.87724 | 7.134612 | -8.69391 | 4.43E-17 | 6.90E-16 | 28.06382 |
| SRGN | -0.70148 | 7.031695 | -8.69101 | 4.53E-17 | 7.05E-16 | 28.04196 |
| GALNT2 | -0.6451 | 4.854538 | -8.66985 | 5.33E-17 | 8.15E-16 | 27.88229 |
| TGFA | -1.01085 | 5.163136 | -8.63943 | 6.73E-17 | 1.01E-15 | 27.65325 |
| ICAM1 | -0.72324 | 5.655362 | -8.62661 | 7.43E-17 | 1.11E-15 | 27.55692 |
| SLC25A24 | -0.62324 | 3.940374 | -8.62608 | 7.46E-17 | 1.11E-15 | 27.55296 |
| ACTR3 | -0.66606 | 6.482003 | -8.61157 | 8.33E-17 | 1.23E-15 | 27.44404 |
| PHAX | -0.59716 | 4.751255 | -8.60058 | 9.06E-17 | 1.33E-15 | 27.36166 |
| JAK1 | -0.59755 | 6.064391 | -8.58525 | 1.02E-16 | 1.48E-15 | 27.24684 |
| MALAT1 | 0.988454 | 6.19533 | 8.550331 | 1.33E-16 | 1.88E-15 | 26.98592 |
| HFE | -0.60063 | 3.428826 | -8.53433 | 1.50E-16 | 2.10E-15 | 26.86664 |
| LYN | -0.66308 | 3.922116 | -8.52643 | 1.59E-16 | 2.21E-15 | 26.80783 |
| BHLHE41 | -0.85381 | 5.481626 | -8.51426 | 1.75E-16 | 2.41E-15 | 26.71721 |
| CD109 | -0.78917 | 3.415031 | -8.5084 | 1.83E-16 | 2.51E-15 | 26.67369 |
| OSBPL10 | -0.64282 | 3.523287 | -8.50657 | 1.85E-16 | 2.54E-15 | 26.66007 |
| DDX21 | -0.61075 | 4.267106 | -8.50547 | 1.87E-16 | 2.56E-15 | 26.65189 |
| TRPA1 | -0.7588 | 2.260857 | -8.50012 | 1.95E-16 | 2.65E-15 | 26.61217 |
| DEGS1 | -0.59106 | 6.34816 | -8.48845 | 2.13E-16 | 2.88E-15 | 26.52555 |
| NFIX | -0.67647 | 4.332249 | -8.48774 | 2.14E-16 | 2.89E-15 | 26.52026 |
| TPM1 | -0.61511 | 8.455679 | -8.48376 | 2.20E-16 | 2.96E-15 | 26.49076 |
| GBE1 | -0.62473 | 4.775515 | -8.4837 | 2.20E-16 | 2.96E-15 | 26.49032 |
| LMCD1 | -0.62395 | 4.741254 | -8.48189 | 2.23E-16 | 3.00E-15 | 26.47689 |
| BICC1 | -0.80153 | 4.586846 | -8.46991 | 2.45E-16 | 3.26E-15 | 26.38809 |
| SF3A1 | -0.66437 | 5.321571 | -8.46212 | 2.59E-16 | 3.44E-15 | 26.33041 |
| ZBTB38 | -0.66556 | 4.728087 | -8.45679 | 2.70E-16 | 3.58E-15 | 26.29095 |
| IL1RAP | -0.58919 | 2.420746 | -8.45195 | 2.80E-16 | 3.70E-15 | 26.25513 |
| ANKH | -0.64281 | 4.669887 | -8.42604 | 3.41E-16 | 4.42E-15 | 26.06379 |
| MGAT2 | -0.65703 | 3.209108 | -8.4167 | 3.65E-16 | 4.73E-15 | 25.99486 |
| SPATS2L | -0.66199 | 6.599037 | -8.41177 | 3.79E-16 | 4.88E-15 | 25.95853 |
| NFIL3 | -0.65143 | 4.746034 | -8.40733 | 3.92E-16 | 5.04E-15 | 25.92581 |
| RBM7 | -0.63686 | 4.269014 | -8.40527 | 3.98E-16 | 5.11E-15 | 25.91065 |
| CRYBG1 | -0.64135 | 3.731692 | -8.39554 | 4.28E-16 | 5.48E-15 | 25.83902 |
| PLEKHA2 | -0.67185 | 4.879671 | -8.37764 | 4.90E-16 | 6.20E-15 | 25.70747 |
| ZNF185 | -0.59333 | 3.194526 | -8.36282 | 5.48E-16 | 6.85E-15 | 25.59861 |
| KIF2A | -0.74545 | 3.355943 | -8.35867 | 5.65E-16 | 7.06E-15 | 25.56823 |
| TMEM123 | -0.67863 | 7.119002 | -8.35221 | 5.93E-16 | 7.38E-15 | 25.52087 |
| EGLN3 | -1.14324 | 8.035127 | -8.35167 | 5.95E-16 | 7.41E-15 | 25.51686 |
| ADAM10 | -0.80376 | 5.517432 | -8.35033 | 6.01E-16 | 7.47E-15 | 25.50708 |
| DDX60 | -0.61951 | 3.823716 | -8.34784 | 6.12E-16 | 7.59E-15 | 25.48886 |
| ADAM9 | -0.70967 | 5.696005 | -8.33994 | 6.50E-16 | 8.02E-15 | 25.43097 |
| CLIC4 | -0.7628 | 7.175313 | -8.33308 | 6.84E-16 | 8.40E-15 | 25.38074 |
| ALDOC | -1.04835 | 5.840776 | -8.32062 | 7.51E-16 | 9.17E-15 | 25.28969 |
| TMEM173 | -0.60405 | 5.418448 | -8.31594 | 7.77E-16 | 9.45E-15 | 25.25547 |
| EGLN1 | -0.63883 | 4.944172 | -8.31121 | 8.05E-16 | 9.76E-15 | 25.22095 |
| ADGRE5 | -0.60733 | 5.337733 | -8.29886 | 8.83E-16 | 1.06E-14 | 25.13081 |
| TEX2 | -0.59675 | 3.876973 | -8.29114 | 9.35E-16 | 1.12E-14 | 25.0745 |
| VSIR | -0.59666 | 5.426719 | -8.28711 | 9.63E-16 | 1.15E-14 | 25.04518 |
| VSIG4 | -0.90156 | 4.391224 | -8.28665 | 9.67E-16 | 1.15E-14 | 25.04184 |
| TYMS | -0.66507 | 5.172201 | -8.27741 | 1.04E-15 | 1.22E-14 | 24.97451 |
| CNKSR3 | -0.69174 | 4.3232 | -8.27507 | 1.05E-15 | 1.24E-14 | 24.9575 |
| BICD1 | -0.58857 | 3.702901 | -8.27025 | 1.09E-15 | 1.28E-14 | 24.92243 |
| PJA2 | -0.58626 | 5.624377 | -8.26193 | 1.16E-15 | 1.36E-14 | 24.86197 |
| NRP1 | -0.76505 | 6.795983 | -8.24078 | 1.36E-15 | 1.57E-14 | 24.70838 |
| BIRC3 | -0.89365 | 5.172663 | -8.23771 | 1.39E-15 | 1.61E-14 | 24.68609 |
| GLIPR1 | -0.72493 | 4.105776 | -8.2309 | 1.46E-15 | 1.68E-14 | 24.63676 |
| OAS1 | -0.6021 | 5.412418 | -8.20286 | 1.80E-15 | 2.03E-14 | 24.43379 |
| CAVIN1 | -0.62375 | 6.854154 | -8.20095 | 1.82E-15 | 2.06E-14 | 24.41998 |
| PTPRF | -0.61649 | 5.514903 | -8.19828 | 1.86E-15 | 2.09E-14 | 24.40074 |
| CDH2 | -0.89692 | 4.654935 | -8.19069 | 1.97E-15 | 2.20E-14 | 24.34593 |
| THBD | -0.68608 | 3.656927 | -8.16441 | 2.39E-15 | 2.63E-14 | 24.15643 |
| HSPA8 | -0.63762 | 8.907922 | -8.1586 | 2.49E-15 | 2.73E-14 | 24.11457 |
| DIXDC1 | -0.65786 | 3.753698 | -8.14435 | 2.77E-15 | 3.01E-14 | 24.01205 |
| IFI16 | -0.58809 | 6.056788 | -8.14187 | 2.82E-15 | 3.06E-14 | 23.99424 |
| TGFB1 | -0.59059 | 5.898245 | -8.12692 | 3.14E-15 | 3.39E-14 | 23.88692 |
| B3GALNT1 | -0.65931 | 3.934364 | -8.11812 | 3.35E-15 | 3.60E-14 | 23.82379 |
| MRAS | -0.65467 | 4.089139 | -8.09479 | 3.98E-15 | 4.22E-14 | 23.65667 |
| IL6ST | -0.61879 | 5.679208 | -8.09438 | 3.99E-15 | 4.23E-14 | 23.65375 |
| SMC1A | -0.65395 | 3.952838 | -8.08018 | 4.42E-15 | 4.65E-14 | 23.55227 |
| TMTC2 | -0.60094 | 2.983032 | -8.07029 | 4.76E-15 | 4.96E-14 | 23.48163 |
| RNF11 | -0.59721 | 6.035562 | -8.04991 | 5.52E-15 | 5.68E-14 | 23.33634 |
| TLR3 | -0.93985 | 4.758891 | -8.0378 | 6.03E-15 | 6.16E-14 | 23.25011 |
| NRP2 | -0.68134 | 4.053638 | -8.02832 | 6.46E-15 | 6.56E-14 | 23.18266 |
| SAMHD1 | -0.63226 | 4.785894 | -8.00003 | 7.93E-15 | 7.92E-14 | 22.98186 |
| SRGAP2B | -0.64269 | 3.490975 | -7.99407 | 8.27E-15 | 8.26E-14 | 22.93964 |
| C1QB | -0.88396 | 7.969922 | -7.9853 | 8.82E-15 | 8.72E-14 | 22.87751 |
| GLIS3 | -0.62579 | 3.305351 | -7.96718 | 1.01E-14 | 9.81E-14 | 22.7494 |
| PDIA5 | -0.67164 | 5.471458 | -7.96691 | 1.01E-14 | 9.82E-14 | 22.74744 |
| TM4SF1 | -0.66697 | 6.475757 | -7.94308 | 1.20E-14 | 1.15E-13 | 22.57929 |
| HPCAL1 | -0.67818 | 7.200236 | -7.92238 | 1.39E-14 | 1.31E-13 | 22.43351 |
| CERCAM | -0.70255 | 4.375619 | -7.92 | 1.41E-14 | 1.33E-13 | 22.41676 |
| OAS2 | -0.6464 | 4.042889 | -7.91318 | 1.48E-14 | 1.40E-13 | 22.36887 |
| DSE | -0.5944 | 3.178118 | -7.84355 | 2.44E-14 | 2.23E-13 | 21.88116 |
| RNASE6 | -0.67217 | 4.557022 | -7.8404 | 2.50E-14 | 2.28E-13 | 21.85921 |
| TMED5 | -0.62995 | 5.180723 | -7.81255 | 3.04E-14 | 2.74E-13 | 21.66517 |
| PPP2R3A | -0.6159 | 3.737881 | -7.80625 | 3.18E-14 | 2.85E-13 | 21.62135 |
| AHR | -0.64537 | 4.262293 | -7.80508 | 3.21E-14 | 2.87E-13 | 21.61316 |
| ENO2 | -0.80603 | 6.421456 | -7.78909 | 3.60E-14 | 3.18E-13 | 21.5021 |
| ANXA4 | -0.67691 | 8.802345 | -7.77771 | 3.90E-14 | 3.43E-13 | 21.42314 |
| MAPK1 | -0.73137 | 5.391857 | -7.76914 | 4.14E-14 | 3.63E-13 | 21.3638 |
| LOXL2 | -0.83629 | 5.02537 | -7.76573 | 4.24E-14 | 3.71E-13 | 21.34017 |
| KLF10 | -0.76395 | 5.537051 | -7.74961 | 4.76E-14 | 4.12E-13 | 21.22862 |
| CD68 | -0.80912 | 7.75503 | -7.72829 | 5.53E-14 | 4.73E-13 | 21.08139 |
| STAT1 | -0.7222 | 6.139559 | -7.71778 | 5.96E-14 | 5.07E-13 | 21.00893 |
| MET | -0.60588 | 5.659734 | -7.70095 | 6.71E-14 | 5.67E-13 | 20.89309 |
| WARS | -0.58594 | 6.533505 | -7.69367 | 7.06E-14 | 5.94E-13 | 20.84299 |
| FCGR3A | -0.86625 | 6.28921 | -7.68555 | 7.47E-14 | 6.25E-13 | 20.78724 |
| LY75 | -0.6193 | 2.40564 | -7.66152 | 8.84E-14 | 7.33E-13 | 20.62242 |
| FSTL1 | -0.82166 | 6.837115 | -7.65896 | 9.00E-14 | 7.45E-13 | 20.60489 |
| C1QA | -0.77824 | 7.834134 | -7.652 | 9.46E-14 | 7.79E-13 | 20.55719 |
| EIF4B | -0.61994 | 7.710237 | -7.65064 | 9.55E-14 | 7.86E-13 | 20.54791 |
| SYT11 | -0.59147 | 3.114839 | -7.64841 | 9.70E-14 | 7.96E-13 | 20.53268 |
| SLFN11 | -0.66728 | 4.15804 | -7.62415 | 1.15E-13 | 9.34E-13 | 20.36691 |
| IFI44L | -0.84513 | 3.922543 | -7.62084 | 1.18E-13 | 9.54E-13 | 20.34435 |
| RNASE4 | -0.77802 | 5.354665 | -7.60162 | 1.34E-13 | 1.08E-12 | 20.21331 |
| EIF2S1 | -0.61624 | 5.574992 | -7.58164 | 1.54E-13 | 1.22E-12 | 20.07745 |
| ARL4C | -0.82155 | 4.431037 | -7.54849 | 1.94E-13 | 1.51E-12 | 19.85264 |
| EPSTI1 | -0.70066 | 3.777433 | -7.54769 | 1.96E-13 | 1.52E-12 | 19.84726 |
| MYC | -0.72533 | 5.526003 | -7.53435 | 2.14E-13 | 1.65E-12 | 19.75701 |
| SEMA4B | -0.58615 | 5.073348 | -7.52198 | 2.34E-13 | 1.79E-12 | 19.67345 |
| SPAG4 | -0.6176 | 6.036043 | -7.50813 | 2.57E-13 | 1.96E-12 | 19.58002 |
| PLEKHA3 | -0.58542 | 3.151271 | -7.50708 | 2.59E-13 | 1.97E-12 | 19.57296 |
| SGCB | -0.58944 | 5.287016 | -7.50581 | 2.61E-13 | 1.98E-12 | 19.56439 |
| SERPING1 | -0.77153 | 8.614683 | -7.50402 | 2.64E-13 | 2.00E-12 | 19.55236 |
| CPVL | -0.7598 | 5.861851 | -7.50363 | 2.65E-13 | 2.01E-12 | 19.54974 |
| SGMS2 | -0.60393 | 3.395909 | -7.50116 | 2.70E-13 | 2.04E-12 | 19.53306 |
| FAT1 | -0.61123 | 6.241222 | -7.46506 | 3.46E-13 | 2.56E-12 | 19.29039 |
| HILPDA | -0.98018 | 7.211872 | -7.46326 | 3.50E-13 | 2.59E-12 | 19.27833 |
| IRF1 | -0.63894 | 5.519279 | -7.45663 | 3.66E-13 | 2.70E-12 | 19.23384 |
| ST8SIA4 | -0.72563 | 3.348512 | -7.43858 | 4.15E-13 | 3.02E-12 | 19.11299 |
| EML1 | -0.59504 | 3.633808 | -7.43673 | 4.20E-13 | 3.06E-12 | 19.10061 |
| C1QC | -0.81373 | 7.528273 | -7.43305 | 4.31E-13 | 3.13E-12 | 19.07604 |
| LIPA | -0.63187 | 6.369315 | -7.42814 | 4.45E-13 | 3.23E-12 | 19.04321 |
| TCN2 | -0.75941 | 6.760154 | -7.42795 | 4.46E-13 | 3.23E-12 | 19.04192 |
| FCF1 | -0.62538 | 4.229015 | -7.42673 | 4.50E-13 | 3.26E-12 | 19.03377 |
| CSF1R | -0.67934 | 4.900182 | -7.41648 | 4.82E-13 | 3.47E-12 | 18.96534 |
| ELK3 | -0.64391 | 4.586089 | -7.41256 | 4.95E-13 | 3.55E-12 | 18.93915 |
| LAPTM5 | -0.63257 | 7.215964 | -7.41159 | 4.99E-13 | 3.57E-12 | 18.9327 |
| OPN3 | -0.58894 | 3.877669 | -7.40771 | 5.12E-13 | 3.66E-12 | 18.90683 |
| ITGA3 | -0.61495 | 6.540064 | -7.40767 | 5.12E-13 | 3.66E-12 | 18.9066 |
| PDGFC | -0.59121 | 4.205906 | -7.39913 | 5.43E-13 | 3.86E-12 | 18.84966 |
| HTRA1 | -0.60644 | 7.684241 | -7.36159 | 7.01E-13 | 4.90E-12 | 18.6001 |
| FPR3 | -0.7589 | 3.858857 | -7.36134 | 7.02E-13 | 4.90E-12 | 18.59848 |
| HEG1 | -0.75479 | 4.482007 | -7.35596 | 7.28E-13 | 5.08E-12 | 18.56276 |
| ITGAV | -0.59981 | 5.6511 | -7.3451 | 7.84E-13 | 5.44E-12 | 18.49082 |
| DPP4 | -0.99359 | 5.552933 | -7.34131 | 8.05E-13 | 5.57E-12 | 18.46574 |
| QSOX1 | -0.60515 | 6.207145 | -7.33065 | 8.65E-13 | 5.95E-12 | 18.39524 |
| AIF1 | -0.61247 | 6.252418 | -7.32382 | 9.06E-13 | 6.22E-12 | 18.35012 |
| SGPP2 | -0.74021 | 3.889951 | -7.32332 | 9.09E-13 | 6.23E-12 | 18.34676 |
| C1R | -1.00242 | 6.886137 | -7.32127 | 9.22E-13 | 6.31E-12 | 18.33326 |
| CA9 | -1.19744 | 7.802635 | -7.31983 | 9.31E-13 | 6.36E-12 | 18.3237 |
| QKI | -0.61523 | 5.250414 | -7.31873 | 9.38E-13 | 6.39E-12 | 18.31646 |
| ALOX5AP | -0.64999 | 4.250875 | -7.29827 | 1.08E-12 | 7.27E-12 | 18.18157 |
| PLCB1 | -0.68062 | 3.599316 | -7.29068 | 1.13E-12 | 7.61E-12 | 18.13157 |
| MRC1 | -0.71538 | 2.427971 | -7.28732 | 1.16E-12 | 7.77E-12 | 18.10942 |
| FOLR2 | -0.75617 | 4.568619 | -7.28722 | 1.16E-12 | 7.77E-12 | 18.10877 |
| ARHGAP29 | -0.59123 | 4.738226 | -7.27358 | 1.27E-12 | 8.49E-12 | 18.0191 |
| CFH | -0.90816 | 4.547371 | -7.26753 | 1.32E-12 | 8.82E-12 | 17.97942 |
| ITGB2 | -0.66061 | 5.935697 | -7.25258 | 1.46E-12 | 9.67E-12 | 17.88136 |
| SPARC | -0.70806 | 10.89436 | -7.21639 | 1.87E-12 | 1.21E-11 | 17.64467 |
| CD86 | -0.58897 | 3.090677 | -7.20502 | 2.01E-12 | 1.30E-11 | 17.57049 |
| TRABD2B | -0.62966 | 3.732998 | -7.19582 | 2.14E-12 | 1.38E-11 | 17.51053 |
| AXL | -0.62618 | 4.805389 | -7.15625 | 2.79E-12 | 1.77E-11 | 17.25347 |
| SEL1L3 | -0.63439 | 5.637296 | -7.15336 | 2.84E-12 | 1.80E-11 | 17.23472 |
| NLGN1 | -0.67448 | 2.849915 | -7.15023 | 2.90E-12 | 1.84E-11 | 17.21446 |
| HLA-DRA | -0.69412 | 10.24604 | -7.14112 | 3.08E-12 | 1.94E-11 | 17.15551 |
| CD14 | -0.66264 | 6.148798 | -7.14013 | 3.10E-12 | 1.95E-11 | 17.14906 |
| CAVIN3 | -0.61117 | 5.649534 | -7.13408 | 3.23E-12 | 2.03E-11 | 17.10995 |
| ITPR3 | -0.62797 | 3.639497 | -7.1335 | 3.24E-12 | 2.03E-11 | 17.10618 |
| SALL1 | -0.67837 | 4.134052 | -7.12849 | 3.35E-12 | 2.10E-11 | 17.07382 |
| EHBP1 | -0.60304 | 4.615234 | -7.12735 | 3.38E-12 | 2.11E-11 | 17.06646 |
| ITGB3 | -0.63312 | 2.801103 | -7.11183 | 3.74E-12 | 2.32E-11 | 16.96628 |
| TM4SF18 | -0.80672 | 5.412334 | -7.09801 | 4.10E-12 | 2.53E-11 | 16.87721 |
| TMCC1 | -0.71772 | 5.781684 | -7.09782 | 4.10E-12 | 2.53E-11 | 16.87599 |
| FSCN1 | -0.60718 | 5.293175 | -7.09221 | 4.26E-12 | 2.62E-11 | 16.83987 |
| EHD2 | -0.64339 | 6.614074 | -7.08959 | 4.33E-12 | 2.66E-11 | 16.82303 |
| VCAM1 | -1.00518 | 7.438703 | -7.08889 | 4.35E-12 | 2.67E-11 | 16.81855 |
| TNFRSF11B | -0.84646 | 4.122316 | -7.08617 | 4.43E-12 | 2.72E-11 | 16.80104 |
| MSR1 | -0.76099 | 4.411397 | -7.0813 | 4.58E-12 | 2.80E-11 | 16.76977 |
| CDH6 | -0.88017 | 5.614927 | -7.08053 | 4.60E-12 | 2.81E-11 | 16.76478 |
| TRIM22 | -0.6076 | 5.578676 | -7.06237 | 5.18E-12 | 3.15E-11 | 16.6482 |
| IGFBP6 | -0.72934 | 4.983847 | -7.05892 | 5.30E-12 | 3.22E-11 | 16.6261 |
| CD44 | -0.67618 | 5.651549 | -7.04698 | 5.74E-12 | 3.47E-11 | 16.54961 |
| CD302 | -0.58955 | 3.15381 | -7.03331 | 6.27E-12 | 3.77E-11 | 16.46224 |
| VWA1 | -0.68229 | 6.413405 | -7.02929 | 6.44E-12 | 3.86E-11 | 16.43653 |
| LOX | -1.13137 | 5.577057 | -7.02359 | 6.69E-12 | 4.00E-11 | 16.40016 |
| AMD1 | -0.58592 | 5.461467 | -7.01164 | 7.23E-12 | 4.29E-11 | 16.32391 |
| SMIM3 | -0.83186 | 4.663588 | -7.00395 | 7.60E-12 | 4.49E-11 | 16.27493 |
| CAV1 | -0.62877 | 6.851705 | -7.00297 | 7.65E-12 | 4.51E-11 | 16.26866 |
| JUN | -0.61015 | 6.637505 | -6.99315 | 8.16E-12 | 4.79E-11 | 16.2062 |
| SOD2 | -0.69438 | 9.096286 | -6.98804 | 8.44E-12 | 4.94E-11 | 16.1737 |
| SMAD4 | -0.61436 | 4.745324 | -6.98411 | 8.66E-12 | 5.07E-11 | 16.14873 |
| IFIT1 | -0.59385 | 5.093369 | -6.97683 | 9.08E-12 | 5.30E-11 | 16.10252 |
| CD163 | -0.77188 | 5.172333 | -6.96415 | 9.86E-12 | 5.73E-11 | 16.02208 |
| PTPRU | -0.66388 | 3.419905 | -6.96014 | 1.01E-11 | 5.86E-11 | 15.99669 |
| TYMP | -0.64675 | 5.996949 | -6.94611 | 1.11E-11 | 6.39E-11 | 15.90786 |
| RGL1 | -0.59166 | 4.431705 | -6.93925 | 1.16E-11 | 6.66E-11 | 15.8645 |
| CADM1 | -0.61567 | 3.708567 | -6.93742 | 1.17E-11 | 6.73E-11 | 15.85294 |
| PERP | -0.62699 | 4.746099 | -6.93088 | 1.22E-11 | 6.99E-11 | 15.81164 |
| S100A9 | -0.78791 | 5.361734 | -6.92504 | 1.27E-11 | 7.25E-11 | 15.77478 |
| PLSCR4 | -0.62585 | 3.985445 | -6.90923 | 1.41E-11 | 7.97E-11 | 15.67511 |
| GIMAP6 | -0.66327 | 4.61407 | -6.90452 | 1.45E-11 | 8.20E-11 | 15.64549 |
| P3H2 | -0.68061 | 5.938373 | -6.90188 | 1.48E-11 | 8.33E-11 | 15.62889 |
| GALNT14 | -0.79226 | 7.089233 | -6.89929 | 1.50E-11 | 8.46E-11 | 15.61261 |
| BCL9L | -0.60209 | 4.201734 | -6.89911 | 1.50E-11 | 8.47E-11 | 15.61148 |
| ANGPTL4 | -1.07147 | 8.555457 | -6.89665 | 1.53E-11 | 8.60E-11 | 15.59595 |
| ETS1 | -0.67983 | 6.138599 | -6.89659 | 1.53E-11 | 8.60E-11 | 15.59559 |
| TNC | -0.88955 | 4.331055 | -6.8809 | 1.69E-11 | 9.46E-11 | 15.49708 |
| C1orf162 | -0.60871 | 5.097205 | -6.86131 | 1.92E-11 | 1.06E-10 | 15.37426 |
| CP | -1.4735 | 6.753377 | -6.85931 | 1.94E-11 | 1.08E-10 | 15.36174 |
| EPHA2 | -0.59362 | 4.064809 | -6.85309 | 2.02E-11 | 1.12E-10 | 15.32284 |
| GLUL | -0.62247 | 7.862165 | -6.84916 | 2.07E-11 | 1.14E-10 | 15.29827 |
| FOXJ3 | -0.69678 | 5.020531 | -6.84466 | 2.13E-11 | 1.18E-10 | 15.27018 |
| SLCO2B1 | -0.67638 | 4.85457 | -6.84417 | 2.14E-11 | 1.18E-10 | 15.26711 |
| STC1 | -0.79589 | 5.583584 | -6.84395 | 2.14E-11 | 1.18E-10 | 15.26574 |
| LY6E | -0.81492 | 7.880905 | -6.83819 | 2.22E-11 | 1.22E-10 | 15.22977 |
| PLAUR | -0.62459 | 4.069973 | -6.838 | 2.23E-11 | 1.22E-10 | 15.22859 |
| MS4A6A | -0.67596 | 6.550621 | -6.82926 | 2.35E-11 | 1.29E-10 | 15.17402 |
| HLA-DPB1 | -0.66827 | 8.616914 | -6.82822 | 2.37E-11 | 1.30E-10 | 15.16757 |
| AKAP12 | -0.80329 | 4.691974 | -6.80125 | 2.82E-11 | 1.52E-10 | 14.99968 |
| PIK3R1 | -0.63135 | 4.423932 | -6.79837 | 2.87E-11 | 1.54E-10 | 14.98181 |
| FZD1 | -0.61334 | 4.644668 | -6.79711 | 2.89E-11 | 1.55E-10 | 14.97394 |
| MS4A4A | -0.65693 | 4.179362 | -6.77202 | 3.39E-11 | 1.81E-10 | 14.81838 |
| HERC3 | -0.58661 | 5.233223 | -6.76972 | 3.44E-11 | 1.83E-10 | 14.80415 |
| MFAP3 | -0.64396 | 3.913024 | -6.76963 | 3.44E-11 | 1.83E-10 | 14.8036 |
| PMEPA1 | -0.65998 | 4.954081 | -6.76629 | 3.52E-11 | 1.87E-10 | 14.78289 |
| NPIPB12 | 0.62646 | 4.195979 | 6.746828 | 3.98E-11 | 2.09E-10 | 14.66263 |
| C3AR1 | -0.61123 | 3.783919 | -6.74199 | 4.10E-11 | 2.15E-10 | 14.63278 |
| RASAL1 | -0.71288 | 3.152988 | -6.7398 | 4.16E-11 | 2.18E-10 | 14.61926 |
| CXCL12 | -0.748 | 5.177597 | -6.72531 | 4.56E-11 | 2.38E-10 | 14.52998 |
| THBS1 | -0.70142 | 6.13503 | -6.71969 | 4.73E-11 | 2.46E-10 | 14.49543 |
| SYNPO | -0.66205 | 6.384416 | -6.71454 | 4.88E-11 | 2.53E-10 | 14.46377 |
| SGCE | -0.62939 | 4.63211 | -6.6847 | 5.90E-11 | 3.01E-10 | 14.28063 |
| FCGR3B | -0.61806 | 1.970234 | -6.68193 | 6.00E-11 | 3.05E-10 | 14.2637 |
| PECAM1 | -0.72422 | 6.835917 | -6.6734 | 6.33E-11 | 3.21E-10 | 14.21149 |
| SPP1 | -0.75381 | 10.00579 | -6.66636 | 6.62E-11 | 3.34E-10 | 14.16843 |
| TMEM200A | -0.70181 | 4.091815 | -6.66451 | 6.69E-11 | 3.38E-10 | 14.15717 |
| DEPP1 | -0.65178 | 7.50751 | -6.66225 | 6.79E-11 | 3.42E-10 | 14.14333 |
| CITED2 | -0.66732 | 6.134117 | -6.61889 | 8.91E-11 | 4.41E-10 | 13.87927 |
| BTBD16 | -0.60294 | 2.094434 | -6.5953 | 1.03E-10 | 5.08E-10 | 13.7362 |
| SLC16A4 | -0.69861 | 5.651288 | -6.58872 | 1.07E-10 | 5.28E-10 | 13.69637 |
| VNN2 | -0.60928 | 2.492953 | -6.56658 | 1.23E-10 | 6.01E-10 | 13.56261 |
| ITM2A | -0.72924 | 4.658234 | -6.565 | 1.25E-10 | 6.06E-10 | 13.55311 |
| SCARA3 | -0.71301 | 2.608416 | -6.56432 | 1.25E-10 | 6.08E-10 | 13.549 |
| TLN2 | -0.61612 | 3.960507 | -6.56198 | 1.27E-10 | 6.16E-10 | 13.53487 |
| HGF | -0.65512 | 2.514363 | -6.56004 | 1.28E-10 | 6.23E-10 | 13.52315 |
| PROS1 | -0.64717 | 5.66798 | -6.55978 | 1.29E-10 | 6.23E-10 | 13.52164 |
| CYBB | -0.76827 | 4.15193 | -6.55771 | 1.30E-10 | 6.31E-10 | 13.50913 |
| SOX9 | -0.73236 | 3.977121 | -6.54687 | 1.39E-10 | 6.70E-10 | 13.44385 |
| NMB | -0.7281 | 4.505449 | -6.5433 | 1.42E-10 | 6.84E-10 | 13.42239 |
| SYTL2 | -0.76712 | 5.261062 | -6.54252 | 1.43E-10 | 6.87E-10 | 13.4177 |
| IFITM1 | -0.59509 | 7.457183 | -6.52574 | 1.59E-10 | 7.57E-10 | 13.31688 |
| FKBP10 | -0.73968 | 6.211557 | -6.51936 | 1.65E-10 | 7.86E-10 | 13.27858 |
| PRSS23 | -0.61197 | 6.389334 | -6.51652 | 1.68E-10 | 7.99E-10 | 13.2616 |
| HLA-DOA | -0.74022 | 4.566265 | -6.51314 | 1.72E-10 | 8.14E-10 | 13.2413 |
| TMEM176B | -0.74808 | 9.036735 | -6.50947 | 1.76E-10 | 8.31E-10 | 13.21935 |
| NSUN5P1 | 0.628025 | 3.153147 | 6.503648 | 1.82E-10 | 8.58E-10 | 13.1845 |
| CD24 | -0.64479 | 10.00317 | -6.49618 | 1.91E-10 | 8.96E-10 | 13.13985 |
| CFI | -0.60367 | 6.29409 | -6.46212 | 2.35E-10 | 1.09E-09 | 12.93665 |
| CHST15 | -0.64597 | 4.363268 | -6.45919 | 2.39E-10 | 1.10E-09 | 12.91927 |
| MRC2 | -0.61147 | 3.653113 | -6.44205 | 2.66E-10 | 1.21E-09 | 12.8174 |
| TNFAIP6 | -1.06417 | 4.858141 | -6.43046 | 2.85E-10 | 1.30E-09 | 12.74863 |
| SLC8A1 | -0.59649 | 2.42344 | -6.41663 | 3.10E-10 | 1.41E-09 | 12.66677 |
| NEDD9 | -0.64525 | 5.11034 | -6.41249 | 3.18E-10 | 1.44E-09 | 12.64226 |
| FAM110C | -0.67497 | 4.093862 | -6.41155 | 3.20E-10 | 1.45E-09 | 12.63672 |
| HLA-DRB1 | -0.62047 | 8.96254 | -6.39499 | 3.54E-10 | 1.59E-09 | 12.53892 |
| LCP1 | -0.64094 | 5.404584 | -6.39247 | 3.59E-10 | 1.61E-09 | 12.52406 |
| FHL2 | -0.73322 | 5.017755 | -6.38559 | 3.74E-10 | 1.67E-09 | 12.48354 |
| PNMA2 | -0.72064 | 4.12646 | -6.37696 | 3.95E-10 | 1.76E-09 | 12.43275 |
| CTSS | -0.75311 | 6.385626 | -6.36789 | 4.17E-10 | 1.85E-09 | 12.37939 |
| LYVE1 | -0.60409 | 3.143061 | -6.36694 | 4.19E-10 | 1.86E-09 | 12.37379 |
| COL8A1 | -0.72655 | 4.412833 | -6.35361 | 4.54E-10 | 2.00E-09 | 12.29555 |
| FIBIN | -0.68854 | 3.228783 | -6.33392 | 5.12E-10 | 2.24E-09 | 12.18019 |
| MYADM | -0.61277 | 6.030361 | -6.31744 | 5.65E-10 | 2.46E-09 | 12.08388 |
| ABI3BP | -0.87389 | 4.895758 | -6.31323 | 5.80E-10 | 2.52E-09 | 12.05935 |
| ASMTL-AS1 | 0.727905 | 2.0904 | 6.308251 | 5.97E-10 | 2.59E-09 | 12.03029 |
| ALOX5 | -0.67987 | 4.251849 | -6.29023 | 6.66E-10 | 2.86E-09 | 11.92535 |
| FGL2 | -0.63898 | 4.523728 | -6.28537 | 6.85E-10 | 2.94E-09 | 11.89709 |
| GPC6 | -0.64858 | 3.469205 | -6.27878 | 7.13E-10 | 3.05E-09 | 11.85882 |
| UTRN | -0.60013 | 4.648356 | -6.26436 | 7.77E-10 | 3.31E-09 | 11.77519 |
| GJA1 | -0.69178 | 5.839623 | -6.25518 | 8.21E-10 | 3.48E-09 | 11.72201 |
| GUCY1A1 | -0.60668 | 4.644427 | -6.25384 | 8.27E-10 | 3.51E-09 | 11.71424 |
| CALCRL | -0.7275 | 4.829827 | -6.25066 | 8.43E-10 | 3.57E-09 | 11.69589 |
| LAMC2 | -0.71444 | 1.695283 | -6.2394 | 9.02E-10 | 3.80E-09 | 11.63082 |
| VCAN | -0.88977 | 5.562917 | -6.23256 | 9.39E-10 | 3.95E-09 | 11.59129 |
| CPE | -0.79079 | 6.67958 | -6.22432 | 9.86E-10 | 4.14E-09 | 11.54377 |
| CPT1B | 0.716361 | 3.137299 | 6.200924 | 1.13E-09 | 4.71E-09 | 11.40918 |
| RNF128 | -0.6725 | 4.635095 | -6.19398 | 1.18E-09 | 4.90E-09 | 11.36934 |
| ZNF395 | -0.62881 | 6.674314 | -6.18961 | 1.21E-09 | 5.01E-09 | 11.34423 |
| FCGR2B | -0.66666 | 3.525574 | -6.18116 | 1.27E-09 | 5.25E-09 | 11.29581 |
| EGFR | -0.59533 | 5.170501 | -6.17877 | 1.29E-09 | 5.32E-09 | 11.2821 |
| CD93 | -0.666 | 5.36757 | -6.16758 | 1.38E-09 | 5.66E-09 | 11.21808 |
| BICDL2 | -0.62472 | 2.088083 | -6.1663 | 1.39E-09 | 5.70E-09 | 11.21079 |
| SPSB1 | -0.58676 | 4.077105 | -6.16555 | 1.40E-09 | 5.73E-09 | 11.20648 |
| IGFBP3 | -0.78674 | 9.912162 | -6.16097 | 1.43E-09 | 5.88E-09 | 11.18029 |
| TGFBR2 | -0.61202 | 6.00815 | -6.16072 | 1.44E-09 | 5.88E-09 | 11.17887 |
| RHOB | -0.68139 | 8.314715 | -6.15557 | 1.48E-09 | 6.05E-09 | 11.14946 |
| PDE6B | -0.59109 | 3.322809 | -6.13907 | 1.63E-09 | 6.63E-09 | 11.05541 |
| TUSC3 | -0.62435 | 4.922993 | -6.13554 | 1.67E-09 | 6.76E-09 | 11.03531 |
| NDUFA4L2 | -1.04367 | 10.08515 | -6.13501 | 1.67E-09 | 6.78E-09 | 11.03228 |
| KRT80 | -0.61317 | 1.928784 | -6.13453 | 1.68E-09 | 6.80E-09 | 11.02958 |
| APOL1 | -0.75244 | 6.472687 | -6.12269 | 1.80E-09 | 7.26E-09 | 10.96225 |
| MXRA7 | -0.61928 | 6.95776 | -6.10975 | 1.94E-09 | 7.79E-09 | 10.8888 |
| F8 | -0.58807 | 4.363005 | -6.10277 | 2.02E-09 | 8.09E-09 | 10.84925 |
| DUSP1 | -0.62017 | 8.881341 | -6.07338 | 2.40E-09 | 9.51E-09 | 10.68305 |
| DDIT4L | -0.81656 | 2.98678 | -6.04938 | 2.76E-09 | 1.09E-08 | 10.54786 |
| LTBP1 | -0.65477 | 3.795804 | -6.04047 | 2.90E-09 | 1.14E-08 | 10.49782 |
| PILRB | 0.705635 | 3.606479 | 6.038869 | 2.93E-09 | 1.15E-08 | 10.48883 |
| SEZ6L2 | -0.67017 | 4.514305 | -6.02726 | 3.13E-09 | 1.22E-08 | 10.42372 |
| B3GNT4 | -0.59074 | 2.414277 | -6.01449 | 3.37E-09 | 1.31E-08 | 10.35221 |
| SLC2A3 | -0.65179 | 5.330436 | -6.01291 | 3.40E-09 | 1.32E-08 | 10.34338 |
| IRX3 | -0.69653 | 5.376283 | -5.99481 | 3.78E-09 | 1.46E-08 | 10.24229 |
| HLA-DPA1 | -0.62684 | 8.816614 | -5.98732 | 3.94E-09 | 1.52E-08 | 10.20052 |
| AMACR | -0.64326 | 5.771853 | -5.96739 | 4.42E-09 | 1.69E-08 | 10.08962 |
| PLS1 | -0.65713 | 3.490578 | -5.96351 | 4.52E-09 | 1.72E-08 | 10.06806 |
| EMP1 | -0.63416 | 5.775474 | -5.95381 | 4.78E-09 | 1.82E-08 | 10.01422 |
| HLA-DQA1 | -0.79837 | 7.053357 | -5.95049 | 4.87E-09 | 1.85E-08 | 9.995824 |
| PCDHGB7 | -0.74512 | 2.733022 | -5.9441 | 5.05E-09 | 1.91E-08 | 9.960467 |
| TFPI | -0.76941 | 6.126946 | -5.93215 | 5.41E-09 | 2.04E-08 | 9.894307 |
| MT2A | -0.71918 | 7.736877 | -5.92807 | 5.54E-09 | 2.09E-08 | 9.871779 |
| TIMP1 | -0.59258 | 9.071167 | -5.92428 | 5.66E-09 | 2.13E-08 | 9.850883 |
| TSC22D3 | -0.60664 | 7.5245 | -5.91577 | 5.94E-09 | 2.23E-08 | 9.803888 |
| PGBD5 | -0.64963 | 2.463948 | -5.9148 | 5.97E-09 | 2.24E-08 | 9.798569 |
| HAVCR1 | -1.01937 | 4.424076 | -5.90532 | 6.31E-09 | 2.35E-08 | 9.746335 |
| GPR34 | -0.58631 | 3.220038 | -5.89984 | 6.50E-09 | 2.42E-08 | 9.716186 |
| TREM1 | -0.61389 | 1.966097 | -5.89264 | 6.78E-09 | 2.52E-08 | 9.676592 |
| FOLR1 | -0.81502 | 5.724186 | -5.88598 | 7.04E-09 | 2.61E-08 | 9.639994 |
| CTHRC1 | -0.82918 | 3.9802 | -5.86372 | 7.98E-09 | 2.92E-08 | 9.518003 |
| NETO2 | -0.61078 | 4.851822 | -5.85987 | 8.16E-09 | 2.98E-08 | 9.496951 |
| FBLN7 | -0.59735 | 2.39048 | -5.85428 | 8.42E-09 | 3.07E-08 | 9.466372 |
| NR1H4 | -0.61641 | 4.688415 | -5.85267 | 8.50E-09 | 3.10E-08 | 9.457621 |
| F2RL1 | -0.60855 | 4.192682 | -5.84113 | 9.07E-09 | 3.29E-08 | 9.394618 |
| ITGB8 | -0.65716 | 4.17968 | -5.82111 | 1.02E-08 | 3.65E-08 | 9.285603 |
| STC2 | -0.66179 | 4.902538 | -5.80658 | 1.10E-08 | 3.95E-08 | 9.206714 |
| FGF11 | -0.70486 | 4.047566 | -5.80623 | 1.10E-08 | 3.95E-08 | 9.204813 |
| NEAT1 | 0.757532 | 7.874093 | 5.801406 | 1.13E-08 | 4.05E-08 | 9.178645 |
| CCND1 | -0.63186 | 7.636151 | -5.79985 | 1.14E-08 | 4.09E-08 | 9.170194 |
| SLC1A1 | -0.63693 | 4.217096 | -5.79726 | 1.16E-08 | 4.14E-08 | 9.156184 |
| PODXL | -0.67968 | 5.784788 | -5.76509 | 1.39E-08 | 4.90E-08 | 8.982315 |
| SULF1 | -0.64458 | 4.770149 | -5.73933 | 1.60E-08 | 5.61E-08 | 8.84374 |
| TSPAN18 | -0.58882 | 4.997502 | -5.72635 | 1.72E-08 | 6.00E-08 | 8.774117 |
| FMOD | -0.76312 | 3.035211 | -5.71311 | 1.85E-08 | 6.44E-08 | 8.703224 |
| CDH11 | -0.62867 | 3.933418 | -5.70128 | 1.98E-08 | 6.83E-08 | 8.640069 |
| PTPRC | -0.59079 | 4.256858 | -5.69638 | 2.03E-08 | 7.00E-08 | 8.613914 |
| HAGHL | 0.623207 | 2.171934 | 5.681603 | 2.21E-08 | 7.57E-08 | 8.535171 |
| C1S | -0.83186 | 6.729822 | -5.68039 | 2.22E-08 | 7.61E-08 | 8.528722 |
| IER3 | -0.65137 | 5.864392 | -5.67834 | 2.25E-08 | 7.69E-08 | 8.517805 |
| ITIH5 | -0.66684 | 4.582491 | -5.65182 | 2.60E-08 | 8.83E-08 | 8.377069 |
| F13A1 | -0.77444 | 3.797632 | -5.64332 | 2.72E-08 | 9.22E-08 | 8.332044 |
| GALNT9 | -0.82525 | 2.656921 | -5.62861 | 2.95E-08 | 9.95E-08 | 8.2543 |
| CHI3L2 | -0.66181 | 2.154312 | -5.62337 | 3.04E-08 | 1.02E-07 | 8.22667 |
| TMEM37 | -0.6244 | 6.588748 | -5.60091 | 3.43E-08 | 1.15E-07 | 8.10846 |
| FOLH1 | -0.60102 | 3.969096 | -5.5934 | 3.58E-08 | 1.19E-07 | 8.069015 |
| TEK | -0.58949 | 3.169406 | -5.59245 | 3.60E-08 | 1.20E-07 | 8.064052 |
| ADGRL4 | -0.61206 | 5.270964 | -5.58951 | 3.65E-08 | 1.21E-07 | 8.048583 |
| HHATL | 0.649428 | 0.439222 | 5.589035 | 3.66E-08 | 1.22E-07 | 8.04611 |
| DCDC2 | -0.62632 | 2.96015 | -5.57301 | 4.00E-08 | 1.32E-07 | 7.962187 |
| APLNR | -0.70738 | 4.225094 | -5.57226 | 4.01E-08 | 1.33E-07 | 7.958226 |
| COL23A1 | -0.84906 | 6.101257 | -5.56516 | 4.17E-08 | 1.37E-07 | 7.921125 |
| MUC1 | -0.76825 | 5.70315 | -5.531 | 5.02E-08 | 1.63E-07 | 7.743144 |
| ENPEP | -0.73067 | 6.375205 | -5.51413 | 5.49E-08 | 1.78E-07 | 7.655594 |
| TIPARP | -0.62465 | 4.234631 | -5.49183 | 6.19E-08 | 1.99E-07 | 7.540244 |
| CD34 | -0.58982 | 6.337584 | -5.48944 | 6.27E-08 | 2.01E-07 | 7.527907 |
| EPHA7 | -0.63081 | 2.882252 | -5.47159 | 6.90E-08 | 2.21E-07 | 7.435923 |
| MXRA5 | -0.61698 | 2.432154 | -5.46819 | 7.02E-08 | 2.24E-07 | 7.418404 |
| SBSPON | -0.62581 | 3.076485 | -5.46744 | 7.05E-08 | 2.25E-07 | 7.414549 |
| CDH13 | -0.64208 | 5.257569 | -5.4562 | 7.49E-08 | 2.38E-07 | 7.356813 |
| TMEM176A | -0.68451 | 8.928672 | -5.45545 | 7.52E-08 | 2.39E-07 | 7.352929 |
| FMO2 | -0.78525 | 3.320996 | -5.45311 | 7.61E-08 | 2.42E-07 | 7.340919 |
| SLC39A14 | -0.60314 | 6.102395 | -5.44355 | 8.01E-08 | 2.53E-07 | 7.291941 |
| ERRFI1 | -0.7162 | 6.073219 | -5.43135 | 8.55E-08 | 2.69E-07 | 7.229491 |
| CFB | -0.65595 | 6.467933 | -5.42478 | 8.85E-08 | 2.78E-07 | 7.195916 |
| NAPSB | -0.59641 | 3.74737 | -5.42092 | 9.03E-08 | 2.84E-07 | 7.176182 |
| SCD5 | -0.75177 | 3.125241 | -5.41446 | 9.35E-08 | 2.93E-07 | 7.14325 |
| SERPINE2 | -0.66668 | 5.827364 | -5.41312 | 9.41E-08 | 2.95E-07 | 7.136437 |
| LYZ | -0.79724 | 7.117971 | -5.41118 | 9.51E-08 | 2.98E-07 | 7.12651 |
| SEMA5B | -0.70498 | 5.892129 | -5.40881 | 9.63E-08 | 3.01E-07 | 7.114427 |
| HLA-G | -1.0084 | 3.930077 | -5.35898 | 1.25E-07 | 3.84E-07 | 6.861668 |
| HLA-DRB5 | -0.76576 | 6.908559 | -5.3557 | 1.27E-07 | 3.90E-07 | 6.845132 |
| COL15A1 | -0.62323 | 4.573095 | -5.35126 | 1.30E-07 | 3.99E-07 | 6.822715 |
| KMO | -0.70379 | 3.423962 | -5.34515 | 1.35E-07 | 4.11E-07 | 6.791869 |
| SERPINF1 | -0.61856 | 5.528934 | -5.32628 | 1.49E-07 | 4.51E-07 | 6.696927 |
| S1PR1 | -0.59624 | 5.102693 | -5.30014 | 1.70E-07 | 5.13E-07 | 6.565888 |
| FKBP5 | -0.68459 | 5.175845 | -5.2986 | 1.72E-07 | 5.17E-07 | 6.558209 |
| PIGY | -0.72467 | 3.545686 | -5.29811 | 1.72E-07 | 5.18E-07 | 6.555716 |
| CLDN1 | -0.62371 | 4.058773 | -5.29199 | 1.78E-07 | 5.34E-07 | 6.525144 |
| KLK4 | 0.587006 | 0.415134 | 5.2869 | 1.82E-07 | 5.48E-07 | 6.499735 |
| KLK1 | 0.854007 | 0.711133 | 5.281609 | 1.87E-07 | 5.63E-07 | 6.473344 |
| SPOCK1 | -0.86396 | 3.435044 | -5.276 | 1.93E-07 | 5.79E-07 | 6.445369 |
| MMP2 | -0.62352 | 4.809746 | -5.26099 | 2.09E-07 | 6.22E-07 | 6.370726 |
| EDN1 | -0.69223 | 4.789779 | -5.25693 | 2.13E-07 | 6.35E-07 | 6.350556 |
| C1QTNF3 | -0.69277 | 3.360939 | -5.25628 | 2.14E-07 | 6.37E-07 | 6.347298 |
| KCNJ16 | -0.62087 | 5.476924 | -5.25452 | 2.16E-07 | 6.42E-07 | 6.338592 |
| AOC1 | -1.20478 | 5.895867 | -5.22304 | 2.54E-07 | 7.48E-07 | 6.182764 |
| MYEOV | -1.01013 | 3.073932 | -5.21904 | 2.59E-07 | 7.63E-07 | 6.162996 |
| SNURF | -0.74547 | 3.24775 | -5.21451 | 2.65E-07 | 7.80E-07 | 6.140686 |
| IGFBP5 | -0.6963 | 7.330968 | -5.21213 | 2.68E-07 | 7.89E-07 | 6.128974 |
| NTM | -0.64042 | 2.566388 | -5.19536 | 2.92E-07 | 8.56E-07 | 6.046419 |
| TGFBI | -1.02826 | 8.383107 | -5.18829 | 3.03E-07 | 8.86E-07 | 6.011705 |
| CYP1B1 | -0.63996 | 3.025718 | -5.18567 | 3.07E-07 | 8.97E-07 | 5.998897 |
| AQP3 | -0.59452 | 5.75791 | -5.11313 | 4.44E-07 | 1.27E-06 | 5.645332 |
| FLT1 | -0.64144 | 6.186386 | -5.1057 | 4.61E-07 | 1.31E-06 | 5.609364 |
| PLIN2 | -0.69905 | 8.879801 | -5.07471 | 5.38E-07 | 1.52E-06 | 5.459918 |
| SERPINE1 | -0.77646 | 6.019959 | -5.04754 | 6.17E-07 | 1.73E-06 | 5.329543 |
| HLA-DQB2 | -0.60434 | 3.506879 | -5.0237 | 6.95E-07 | 1.94E-06 | 5.215691 |
| SOCS3 | -0.58549 | 5.345345 | -5.01268 | 7.34E-07 | 2.05E-06 | 5.163243 |
| TESC | -0.60038 | 3.409693 | -5.00931 | 7.46E-07 | 2.08E-06 | 5.147221 |
| PTPRB | -0.59728 | 4.036763 | -4.98888 | 8.25E-07 | 2.28E-06 | 5.050299 |
| MOXD1 | -0.60002 | 2.067489 | -4.98396 | 8.46E-07 | 2.34E-06 | 5.026996 |
| CDCP1 | -0.60426 | 2.457743 | -4.96346 | 9.36E-07 | 2.57E-06 | 4.930191 |
| UGT2A3 | -0.86805 | 5.177082 | -4.94149 | 1.04E-06 | 2.85E-06 | 4.826844 |
| MAOB | -0.60008 | 6.498357 | -4.89165 | 1.33E-06 | 3.59E-06 | 4.593924 |
| TMEM130 | -0.85942 | 2.725154 | -4.88266 | 1.39E-06 | 3.73E-06 | 4.552148 |
| FOXI1 | 0.624624 | 0.404666 | 4.881475 | 1.40E-06 | 3.75E-06 | 4.546625 |
| MT1F | -0.65287 | 4.815464 | -4.8747 | 1.44E-06 | 3.87E-06 | 4.515212 |
| QPCT | -0.63225 | 3.435729 | -4.85936 | 1.55E-06 | 4.15E-06 | 4.444182 |
| LAMB3 | -0.63454 | 2.55737 | -4.8405 | 1.70E-06 | 4.51E-06 | 4.357095 |
| COL6A3 | -0.619 | 4.199804 | -4.8185 | 1.89E-06 | 4.99E-06 | 4.255984 |
| COL3A1 | -0.65328 | 6.683105 | -4.81399 | 1.93E-06 | 5.09E-06 | 4.23528 |
| MUC20 | 0.722617 | 4.289749 | 4.806082 | 2.01E-06 | 5.28E-06 | 4.199067 |
| ATP6V0D2 | 0.70026 | 0.852019 | 4.79985 | 2.07E-06 | 5.43E-06 | 4.170556 |
| C2 | -0.71054 | 4.864712 | -4.79866 | 2.08E-06 | 5.46E-06 | 4.165107 |
| FBXL16 | -0.72094 | 5.005303 | -4.79811 | 2.09E-06 | 5.47E-06 | 4.162623 |
| PCSK6 | -0.71371 | 5.113657 | -4.77833 | 2.29E-06 | 5.98E-06 | 4.072366 |
| ZACN | 0.606057 | 0.962743 | 4.767438 | 2.42E-06 | 6.29E-06 | 4.02284 |
| SHISA3 | -0.63873 | 1.954447 | -4.7591 | 2.51E-06 | 6.53E-06 | 3.984993 |
| C4B | -0.62705 | 8.653234 | -4.7495 | 2.63E-06 | 6.82E-06 | 3.941461 |
| IL1R2 | -0.76193 | 2.658844 | -4.74737 | 2.66E-06 | 6.89E-06 | 3.931825 |
| KDR | -0.62497 | 5.300049 | -4.74354 | 2.71E-06 | 7.01E-06 | 3.9145 |
| TMEM252 | -0.68947 | 2.90792 | -4.7099 | 3.17E-06 | 8.15E-06 | 3.762846 |
| MXRA8 | -0.63969 | 5.318767 | -4.68008 | 3.65E-06 | 9.29E-06 | 3.629286 |
| RHCG | 0.731285 | 0.669684 | 4.675897 | 3.72E-06 | 9.46E-06 | 3.610596 |
| HLA-DQA2 | -0.78392 | 3.930454 | -4.66485 | 3.92E-06 | 9.93E-06 | 3.561342 |
| PVALB | 0.890053 | 1.124519 | 4.650991 | 4.18E-06 | 1.06E-05 | 3.499707 |
| ATP6V1B1 | 0.628849 | 1.186386 | 4.646293 | 4.27E-06 | 1.08E-05 | 3.478849 |
| IGFBP2 | -0.60948 | 4.492513 | -4.63471 | 4.51E-06 | 1.13E-05 | 3.427509 |
| ATP6V1G3 | 0.588764 | 0.414764 | 4.59078 | 5.52E-06 | 1.37E-05 | 3.233884 |
| TMEM92 | -0.64814 | 2.430007 | -4.58144 | 5.77E-06 | 1.43E-05 | 3.192924 |
| FHL1 | -0.59123 | 6.951835 | -4.57161 | 6.03E-06 | 1.49E-05 | 3.149904 |
| MAL2 | -0.63168 | 3.647142 | -4.50505 | 8.17E-06 | 1.99E-05 | 2.860933 |
| C6orf223 | -0.64244 | 3.785523 | -4.48754 | 8.85E-06 | 2.14E-05 | 2.785558 |
| PCOLCE2 | -0.7368 | 4.656494 | -4.46537 | 9.78E-06 | 2.36E-05 | 2.690481 |
| AIF1L | -0.67376 | 5.162529 | -4.4522 | 1.04E-05 | 2.49E-05 | 2.634228 |
| FLRT3 | -0.62979 | 2.892982 | -4.4375 | 1.11E-05 | 2.65E-05 | 2.571625 |
| C19orf33 | -0.69188 | 6.178336 | -4.43157 | 1.14E-05 | 2.72E-05 | 2.546408 |
| MT1X | -0.68275 | 6.549195 | -4.42429 | 1.18E-05 | 2.80E-05 | 2.515525 |
| PLTP | -0.60425 | 5.594989 | -4.42019 | 1.20E-05 | 2.85E-05 | 2.498159 |
| MT1E | -0.73279 | 6.372164 | -4.41158 | 1.24E-05 | 2.96E-05 | 2.461714 |
| MGST1 | -0.59495 | 7.469383 | -4.36722 | 1.51E-05 | 3.56E-05 | 2.274933 |
| PTGS1 | -0.59217 | 3.694685 | -4.34378 | 1.68E-05 | 3.93E-05 | 2.176964 |
| UGT2B7 | -0.60193 | 6.864544 | -4.34181 | 1.69E-05 | 3.96E-05 | 2.168752 |
| MDK | -0.60714 | 5.612952 | -4.31595 | 1.90E-05 | 4.41E-05 | 2.061313 |
| CXCL10 | -0.61151 | 4.635859 | -4.31451 | 1.91E-05 | 4.44E-05 | 2.055347 |
| DCN | -0.86208 | 4.47225 | -4.29001 | 2.13E-05 | 4.91E-05 | 1.954091 |
| ANPEP | -0.61401 | 6.887218 | -4.2843 | 2.18E-05 | 5.03E-05 | 1.930596 |
| BHMT | -0.73531 | 6.344317 | -4.27753 | 2.24E-05 | 5.17E-05 | 1.902749 |
| VIL1 | -0.6767 | 2.610063 | -4.26657 | 2.35E-05 | 5.40E-05 | 1.857767 |
| GGT1 | -0.5985 | 7.143233 | -4.26536 | 2.37E-05 | 5.42E-05 | 1.85281 |
| SERPINA1 | -0.6708 | 9.472131 | -4.24974 | 2.53E-05 | 5.78E-05 | 1.788871 |
| SLC3A1 | -0.65594 | 8.209008 | -4.22019 | 2.87E-05 | 6.52E-05 | 1.668556 |
| EFEMP1 | -0.66018 | 5.198064 | -4.20671 | 3.04E-05 | 6.89E-05 | 1.613937 |
| APOLD1 | -0.59084 | 5.534921 | -4.20372 | 3.08E-05 | 6.97E-05 | 1.601827 |
| SLC44A4 | -0.59065 | 2.816359 | -4.16661 | 3.61E-05 | 8.09E-05 | 1.452402 |
| PDZK1IP1 | -0.62005 | 8.811681 | -4.0667 | 5.50E-05 | 0.00012 | 1.056256 |
| MCHR1 | -0.59251 | 1.843385 | -4.05504 | 5.77E-05 | 0.000126 | 1.010606 |
| CCL20 | -0.73352 | 3.861519 | -4.03324 | 6.31E-05 | 0.000137 | 0.925578 |
| ACMSD | -0.59371 | 5.171869 | -4.02933 | 6.42E-05 | 0.000139 | 0.91039 |
| TSPAN1 | -0.67771 | 6.128365 | -4.01749 | 6.74E-05 | 0.000146 | 0.864425 |
| CUBN | -0.6782 | 4.730991 | -4.01245 | 6.88E-05 | 0.000148 | 0.844906 |
| CLDN2 | -0.73293 | 5.975625 | -3.94487 | 9.06E-05 | 0.000193 | 0.585409 |
| S100A1 | -0.8208 | 6.260689 | -3.90055 | 0.000108 | 0.000229 | 0.417448 |
| COL1A1 | -0.60494 | 6.579285 | -3.79414 | 0.000165 | 0.00034 | 0.021569 |
| SFRP2 | -0.78309 | 3.173997 | -3.77792 | 0.000176 | 0.000361 | -0.03788 |
| ENPP3 | -0.64974 | 6.192482 | -3.76838 | 0.000183 | 0.000374 | -0.07271 |
| PROM1 | -0.79038 | 3.039839 | -3.70817 | 0.000231 | 0.000465 | -0.29073 |
| SLC34A2 | -0.74078 | 2.596008 | -3.69741 | 0.000241 | 0.000484 | -0.32931 |
| MMP7 | -0.78573 | 5.367226 | -3.69363 | 0.000244 | 0.000491 | -0.34284 |
| NEFL | -0.64815 | 3.040722 | -3.66931 | 0.000268 | 0.000536 | -0.42965 |
| SPON1 | -0.68675 | 3.230026 | -3.60691 | 0.000339 | 0.00067 | -0.64981 |
| C7 | -0.71957 | 3.99573 | -3.58134 | 0.000373 | 0.000733 | -0.73901 |
| MSLN | -0.74589 | 1.765229 | -3.5574 | 0.000408 | 0.000796 | -0.82195 |
| LRP2 | -0.66743 | 5.63534 | -3.53974 | 0.000436 | 0.000847 | -0.88281 |
| LUM | -0.72531 | 4.840573 | -3.50349 | 0.000498 | 0.000961 | -1.00681 |
| GSTA1 | -0.78639 | 7.421442 | -3.49024 | 0.000523 | 0.001005 | -1.05182 |
| SLPI | -0.823 | 3.153732 | -3.46374 | 0.000576 | 0.001101 | -1.14138 |
| MFAP4 | -0.59393 | 3.61761 | -3.46127 | 0.000581 | 0.00111 | -1.14968 |
| PRIMA1 | -0.59349 | 2.607553 | -3.40706 | 0.000707 | 0.001336 | -1.33071 |
| IGKV2-24 | -0.5878 | 2.780737 | -3.39079 | 0.000749 | 0.001412 | -1.38452 |
| REG1A | -0.85366 | 4.33873 | -3.36247 | 0.000828 | 0.001551 | -1.47759 |
| FABP7 | -1.07772 | 5.493138 | -3.35745 | 0.000843 | 0.001578 | -1.49402 |
| FGB | -0.98217 | 2.946774 | -3.34498 | 0.000881 | 0.001644 | -1.5347 |
| CXCL14 | -0.58601 | 7.688211 | -3.32989 | 0.000929 | 0.001727 | -1.58373 |
| IGHA1 | -0.6539 | 7.19574 | -3.23966 | 0.001272 | 0.002319 | -1.87248 |
| C1QL1 | -0.58983 | 3.55447 | -3.19082 | 0.001503 | 0.002708 | -2.02562 |
| PAH | 0.633362 | 2.548797 | 3.168821 | 0.001619 | 0.002904 | -2.09385 |
| PTHLH | -0.65804 | 3.557373 | -3.15572 | 0.001692 | 0.003026 | -2.13427 |
| LTF | -0.59955 | 2.698971 | -3.04112 | 0.002474 | 0.004321 | -2.48099 |
| NPTX2 | -0.61743 | 4.853881 | -2.9043 | 0.003835 | 0.006517 | -2.8787 |
| KRT19 | -0.68394 | 5.40951 | -2.79306 | 0.005411 | 0.009014 | -3.18899 |
| FGG | -0.73143 | 2.227773 | -2.74625 | 0.006234 | 0.010286 | -3.31604 |
| IGHG1 | -0.63913 | 8.162712 | -2.57145 | 0.0104 | 0.016595 | -3.77202 |
