# Supplementary material for: Identification of Prognostic Metabolism-Related Genes in Clear Cell Renal Cell Carcinoma
Source: J Oncol. 2021 Sep 27;2021:2042114. doi: 10.1155/2021/2042114 (PMC8490028; doi:10.1155/2021/2042114)
Supplement: Supplementary Materials — Supplementary Table S1: clinicopathologic parameters of TCGA KIRC dataset. Supplementary Table S2: DEG to clusters. Supplementary Table S3: univar result after limma final. Supplementary Figure S1: flowchart of data collection and analysis in this study. Supplementary Figure S2: the relationship between the cophenetic coefficient and the number of clusters. Supplementary Figure S3: survival analysis of MDK, SGCB, C4orf3, PILRB, IGHG1, IFITM1, MUC20, and KRT80. [file 2042114.f1.zip › 2042114.f1/Supplementary Table S3.docx]

Supplemental Table S3. Univar result after limma final.

| gene | HR | lower.95 | upper.95 | pvalue |
| --- | --- | --- | --- | --- |
| TPI1 | 0.767356 | 0.605904 | 0.97183 | 0.028017 |
| PKM | 0.701745 | 0.549868 | 0.895571 | 0.004423 |
| PGAM1 | 0.693239 | 0.578338 | 0.830966 | 7.40E-05 |
| LRRC42 | 0.677972 | 0.544237 | 0.844569 | 0.000527 |
| PGK1 | 0.680203 | 0.588147 | 0.786667 | 0 |
| LDHA | 0.790873 | 0.683109 | 0.915638 | 0.001694 |
| ENO1 | 0.787432 | 0.674586 | 0.919156 | 0.002461 |
| TAGLN2 | 1.312154 | 1.011762 | 1.701733 | 0.040548 |
| LMAN2 | 0.69511 | 0.546392 | 0.884306 | 0.003066 |
| TCTN3 | 0.578662 | 0.461141 | 0.726133 | 2.00E-06 |
| YWHAQ | 0.68846 | 0.56139 | 0.844291 | 0.000336 |
| TPM3 | 0.712661 | 0.573421 | 0.885711 | 0.002257 |
| PGM1 | 0.720723 | 0.595109 | 0.872851 | 0.000803 |
| ANXA2 | 1.269148 | 1.016448 | 1.584673 | 0.035381 |
| MSH6 | 0.765887 | 0.626248 | 0.936663 | 0.009402 |
| JPT2 | 0.720843 | 0.599574 | 0.866638 | 0.000496 |
| LAPTM4A | 0.656779 | 0.531236 | 0.811989 | 0.000103 |
| DDX50 | 0.674705 | 0.54705 | 0.83215 | 0.000236 |
| ASCC1 | 0.625924 | 0.515394 | 0.760158 | 2.00E-06 |
| TMED2 | 0.743491 | 0.593639 | 0.931169 | 0.009853 |
| RRM1 | 0.726593 | 0.57746 | 0.91424 | 0.006432 |
| PRPS1 | 0.71407 | 0.579259 | 0.880257 | 0.001607 |
| INSIG2 | 0.751839 | 0.623451 | 0.906666 | 0.002831 |
| ACLY | 0.785814 | 0.694912 | 0.888608 | 0.000122 |
| SNX7 | 0.680246 | 0.583877 | 0.792522 | 1.00E-06 |
| S100A16 | 1.290037 | 1.025624 | 1.622618 | 0.029543 |
| SERBP1 | 0.690269 | 0.572157 | 0.832762 | 0.000108 |
| AK4 | 0.863904 | 0.777147 | 0.960347 | 0.006743 |
| RPA1 | 0.675013 | 0.55589 | 0.819665 | 7.30E-05 |
| PPT1 | 0.623472 | 0.518533 | 0.749649 | 1.00E-06 |
| C5orf15 | 0.728487 | 0.614754 | 0.863262 | 0.000254 |
| PLIN3 | 1.336689 | 1.057488 | 1.689606 | 0.015201 |
| CDC42EP4 | 0.730719 | 0.600277 | 0.889506 | 0.001766 |
| RCN1 | 1.385352 | 1.099694 | 1.745212 | 0.005665 |
| MCMBP | 0.678743 | 0.558825 | 0.824394 | 9.40E-05 |
| ERGIC1 | 0.756046 | 0.623716 | 0.916452 | 0.00439 |
| GDI2 | 0.615531 | 0.506031 | 0.748727 | 1.00E-06 |
| ISG20L2 | 0.716383 | 0.590434 | 0.869199 | 0.000722 |
| C4orf3 | 0.5437 | 0.45007 | 0.656809 | 0 |
| MPI | 0.616683 | 0.511715 | 0.743184 | 0 |
| DHRS13 | 0.747373 | 0.61155 | 0.913361 | 0.004433 |
| PIGS | 0.748423 | 0.624761 | 0.896561 | 0.001661 |
| SSR1 | 0.743458 | 0.616209 | 0.896985 | 0.001968 |
| BNIP3 | 0.733415 | 0.642739 | 0.836883 | 4.00E-06 |
| PDK1 | 0.760514 | 0.64752 | 0.893226 | 0.00085 |
| PLSCR1 | 0.82555 | 0.688373 | 0.990062 | 0.038669 |
| CFAP36 | 0.807925 | 0.676537 | 0.964829 | 0.018505 |
| BTG1 | 0.802014 | 0.679493 | 0.946627 | 0.009095 |
| ERLIN1 | 0.688135 | 0.579886 | 0.816591 | 1.90E-05 |
| PDIA4 | 1.266235 | 1.033424 | 1.551493 | 0.022778 |
| G3BP1 | 0.677618 | 0.564243 | 0.813775 | 3.10E-05 |
| NNMT | 1.12268 | 1.005154 | 1.253947 | 0.040258 |
| IFT57 | 0.640049 | 0.53795 | 0.761525 | 0 |
| SKP1 | 0.620506 | 0.525158 | 0.733166 | 0 |
| RIMKLA | 0.645598 | 0.550563 | 0.757038 | 0 |
| FAS | 0.81618 | 0.700673 | 0.950729 | 0.009082 |
| PAM | 0.755912 | 0.640495 | 0.892127 | 0.000932 |
| TOMM20 | 0.584302 | 0.485126 | 0.703754 | 0 |
| HSPA13 | 0.744202 | 0.611765 | 0.90531 | 0.003128 |
| CREG1 | 0.729506 | 0.616522 | 0.863197 | 0.000239 |
| MCFD2 | 0.677795 | 0.571557 | 0.803781 | 8.00E-06 |
| NRAS | 0.681135 | 0.564934 | 0.821238 | 5.70E-05 |
| GALNT1 | 0.809691 | 0.662946 | 0.988918 | 0.038528 |
| ODC1 | 0.768637 | 0.652464 | 0.905494 | 0.001647 |
| MSN | 0.785804 | 0.664596 | 0.929118 | 0.004801 |
| CANX | 0.651982 | 0.55754 | 0.76242 | 0 |
| RALB | 0.645689 | 0.547848 | 0.761004 | 0 |
| ERAP1 | 0.772205 | 0.654374 | 0.911253 | 0.002213 |
| PFKP | 0.837308 | 0.74093 | 0.946224 | 0.004428 |
| ADAM17 | 0.783442 | 0.647926 | 0.947301 | 0.01178 |
| WWTR1 | 0.742175 | 0.645328 | 0.853556 | 2.90E-05 |
| MTDH | 0.731094 | 0.626985 | 0.85249 | 6.40E-05 |
| TMED10 | 0.634648 | 0.530618 | 0.759073 | 1.00E-06 |
| TNFAIP8 | 0.846585 | 0.719461 | 0.99617 | 0.044836 |
| BNIP3L | 0.770448 | 0.679494 | 0.873575 | 4.70E-05 |
| LRP1 | 1.359279 | 1.108654 | 1.66656 | 0.003158 |
| F11R | 0.672571 | 0.56947 | 0.794338 | 3.00E-06 |
| RNF19B | 0.654932 | 0.56308 | 0.761766 | 0 |
| PTPN14 | 0.711746 | 0.594779 | 0.851715 | 0.000205 |
| YIPF5 | 0.661053 | 0.567952 | 0.769416 | 0 |
| CAND1 | 0.674845 | 0.565019 | 0.806017 | 1.40E-05 |
| NPM1 | 0.678091 | 0.56484 | 0.814048 | 3.10E-05 |
| NEK6 | 0.84697 | 0.75721 | 0.947371 | 0.003662 |
| BZW1 | 0.685434 | 0.587166 | 0.800149 | 2.00E-06 |
| LEPROT | 0.657141 | 0.550293 | 0.784735 | 4.00E-06 |
| GPX8 | 1.223941 | 1.02317 | 1.464109 | 0.027069 |
| GALNT18 | 0.747468 | 0.649872 | 0.85972 | 4.60E-05 |
| NDRG1 | 0.799954 | 0.711209 | 0.899774 | 0.000199 |
| RECQL | 0.772729 | 0.653792 | 0.913303 | 0.002499 |
| ANO6 | 0.774283 | 0.661009 | 0.906967 | 0.001524 |
| TMEM45A | 1.174098 | 1.066369 | 1.292711 | 0.001081 |
| TMEM183B | 0.622926 | 0.521441 | 0.744164 | 0 |
| WDR3 | 0.678909 | 0.577563 | 0.798039 | 3.00E-06 |
| EVA1A | 0.791603 | 0.689153 | 0.909283 | 0.00095 |
| PLOD2 | 1.288692 | 1.112051 | 1.493392 | 0.000746 |
| TGOLN2 | 0.703662 | 0.60188 | 0.822656 | 1.00E-05 |
| ESYT2 | 0.697776 | 0.59067 | 0.824303 | 2.30E-05 |
| LRP10 | 0.722291 | 0.617262 | 0.84519 | 5.00E-05 |
| ANTXR2 | 0.837759 | 0.730619 | 0.960611 | 0.011227 |
| APP | 0.636227 | 0.544366 | 0.743588 | 0 |
| KCTD17 | 1.563061 | 1.26279 | 1.934732 | 4.10E-05 |
| NT5E | 0.819514 | 0.706334 | 0.95083 | 0.008668 |
| CMTM3 | 1.287274 | 1.065369 | 1.555398 | 0.008899 |
| TRIP12 | 0.72412 | 0.61264 | 0.855885 | 0.000154 |
| PIK3CB | 0.667877 | 0.561195 | 0.79484 | 5.00E-06 |
| FTO | 0.74302 | 0.640016 | 0.862602 | 9.60E-05 |
| SLC33A1 | 0.648634 | 0.549677 | 0.765407 | 0 |
| EXT1 | 0.752934 | 0.634782 | 0.893076 | 0.00112 |
| DDX58 | 0.728754 | 0.616687 | 0.861186 | 0.000204 |
| GALNT2 | 1.343408 | 1.133408 | 1.592317 | 0.000664 |
| TGFA | 0.822546 | 0.751081 | 0.90081 | 2.50E-05 |
| SLC25A24 | 0.65781 | 0.561705 | 0.770358 | 0 |
| ACTR3 | 0.747917 | 0.646206 | 0.865637 | 9.80E-05 |
| PHAX | 0.616178 | 0.521479 | 0.728073 | 0 |
| JAK1 | 0.677375 | 0.584438 | 0.78509 | 0 |
| MALAT1 | 1.169237 | 1.046436 | 1.306449 | 0.00575 |
| HFE | 0.705682 | 0.593235 | 0.839442 | 8.30E-05 |
| LYN | 0.827886 | 0.705924 | 0.97092 | 0.020184 |
| OSBPL10 | 0.71272 | 0.605805 | 0.838504 | 4.40E-05 |
| DDX21 | 0.7571 | 0.63997 | 0.895669 | 0.001175 |
| TRPA1 | 0.770787 | 0.671544 | 0.884696 | 0.000214 |
| NFIX | 0.776753 | 0.670649 | 0.899643 | 0.000748 |
| TPM1 | 0.814878 | 0.695195 | 0.955164 | 0.011538 |
| GBE1 | 0.778988 | 0.662006 | 0.91664 | 0.002627 |
| LMCD1 | 0.799034 | 0.675882 | 0.944626 | 0.008614 |
| SF3A1 | 0.659789 | 0.569465 | 0.764438 | 0 |
| ZBTB38 | 0.760052 | 0.650983 | 0.887394 | 0.000517 |
| ANKH | 0.748537 | 0.643844 | 0.870255 | 0.000165 |
| MGAT2 | 0.778658 | 0.669574 | 0.905514 | 0.001159 |
| NFIL3 | 0.838178 | 0.716356 | 0.980717 | 0.027598 |
| RBM7 | 0.673917 | 0.579653 | 0.78351 | 0 |
| CRYBG1 | 0.819801 | 0.698727 | 0.961854 | 0.014811 |
| PLEKHA2 | 0.749539 | 0.649706 | 0.864712 | 7.70E-05 |
| ZNF185 | 0.804966 | 0.678069 | 0.95561 | 0.013185 |
| TMEM123 | 0.776482 | 0.66976 | 0.900211 | 0.000798 |
| EGLN3 | 0.898777 | 0.829512 | 0.973826 | 0.009103 |
| ADAM10 | 0.722313 | 0.639822 | 0.815439 | 0 |
| DDX60 | 0.722964 | 0.623851 | 0.837824 | 1.60E-05 |
| ADAM9 | 0.855435 | 0.739077 | 0.990113 | 0.036335 |
| CLIC4 | 0.782598 | 0.696797 | 0.878964 | 3.50E-05 |
| EGLN1 | 0.65028 | 0.562526 | 0.751723 | 0 |
| TEX2 | 0.660585 | 0.554731 | 0.786638 | 3.00E-06 |
| VSIR | 0.82766 | 0.7063 | 0.969873 | 0.019383 |
| VSIG4 | 1.162053 | 1.026365 | 1.31568 | 0.017752 |
| TYMS | 0.806177 | 0.701566 | 0.926385 | 0.00238 |
| CNKSR3 | 0.728068 | 0.637688 | 0.831257 | 3.00E-06 |
| PJA2 | 0.657224 | 0.560648 | 0.770435 | 0 |
| NRP1 | 0.7759 | 0.689424 | 0.873222 | 2.60E-05 |
| THBD | 0.835806 | 0.716429 | 0.975074 | 0.02255 |
| HSPA8 | 0.69473 | 0.600814 | 0.803325 | 1.00E-06 |
| DIXDC1 | 0.716244 | 0.618428 | 0.829532 | 8.00E-06 |
| IFI16 | 1.590051 | 1.265732 | 1.99747 | 6.80E-05 |
| TGFB1 | 1.319678 | 1.077734 | 1.615938 | 0.007265 |
| B3GALNT1 | 0.693852 | 0.59733 | 0.805972 | 2.00E-06 |
| MRAS | 0.787147 | 0.673751 | 0.919629 | 0.002564 |
| IL6ST | 0.713926 | 0.616052 | 0.82735 | 7.00E-06 |
| SMC1A | 0.777861 | 0.669837 | 0.903307 | 0.000991 |
| TMTC2 | 0.674106 | 0.578546 | 0.785449 | 0 |
| RNF11 | 0.63462 | 0.544083 | 0.740223 | 0 |
| TLR3 | 0.763745 | 0.693191 | 0.841481 | 0 |
| SRGAP2B | 0.752129 | 0.648231 | 0.87268 | 0.000173 |
| GLIS3 | 0.733308 | 0.631561 | 0.851446 | 4.70E-05 |
| CERCAM | 1.50269 | 1.281156 | 1.762531 | 1.00E-06 |
| TMED5 | 0.65499 | 0.563933 | 0.760749 | 0 |
| PPP2R3A | 0.630888 | 0.536581 | 0.74177 | 0 |
| AHR | 0.72038 | 0.621766 | 0.834635 | 1.30E-05 |
| ENO2 | 1.200741 | 1.036589 | 1.390889 | 0.014725 |
| ANXA4 | 0.77118 | 0.674084 | 0.882263 | 0.000154 |
| MAPK1 | 0.718393 | 0.632897 | 0.815439 | 0 |
| KLF10 | 0.768901 | 0.6783 | 0.871604 | 4.00E-05 |
| WARS | 0.819025 | 0.692529 | 0.968627 | 0.019682 |
| EIF4B | 0.677017 | 0.584456 | 0.784236 | 0 |
| SYT11 | 0.754959 | 0.642429 | 0.887199 | 0.000642 |
| RNASE4 | 0.833449 | 0.749243 | 0.927119 | 0.000801 |
| EIF2S1 | 0.686235 | 0.595356 | 0.790987 | 0 |
| ARL4C | 1.225167 | 1.084961 | 1.383492 | 0.001057 |
| SEMA4B | 1.34144 | 1.132501 | 1.588928 | 0.000673 |
| PLEKHA3 | 0.629611 | 0.542467 | 0.730754 | 0 |
| SGCB | 0.656606 | 0.573849 | 0.751298 | 0 |
| CPVL | 0.845541 | 0.75228 | 0.950364 | 0.004896 |
| SGMS2 | 0.647822 | 0.555992 | 0.754818 | 0 |
| ST8SIA4 | 0.775216 | 0.681911 | 0.881287 | 1.00E-04 |
| EML1 | 0.653498 | 0.561143 | 0.761054 | 0 |
| LIPA | 0.698841 | 0.607233 | 0.804269 | 1.00E-06 |
| TCN2 | 0.777186 | 0.697719 | 0.865705 | 5.00E-06 |
| FCF1 | 0.727876 | 0.635442 | 0.833755 | 5.00E-06 |
| ELK3 | 0.779785 | 0.675031 | 0.900794 | 0.000726 |
| PDGFC | 0.717513 | 0.613736 | 0.838837 | 3.10E-05 |
| HEG1 | 0.756989 | 0.670993 | 0.854006 | 6.00E-06 |
| ITGAV | 0.747665 | 0.647266 | 0.863637 | 7.70E-05 |
| DPP4 | 0.801453 | 0.737498 | 0.870954 | 0 |
| QSOX1 | 1.418966 | 1.222655 | 1.646796 | 4.00E-06 |
| SGPP2 | 0.735597 | 0.653562 | 0.827929 | 0 |
| C1R | 1.277408 | 1.148214 | 1.421138 | 7.00E-06 |
| QKI | 0.743424 | 0.642301 | 0.860467 | 7.10E-05 |
| PLCB1 | 0.762787 | 0.673868 | 0.863439 | 1.90E-05 |
| MRC1 | 0.789558 | 0.687474 | 0.906801 | 0.000823 |
| ARHGAP29 | 0.827652 | 0.714736 | 0.958408 | 0.011484 |
| CFH | 1.155614 | 1.037941 | 1.286628 | 0.0083 |
| SPARC | 0.828935 | 0.727862 | 0.944043 | 0.004685 |
| TRABD2B | 0.784438 | 0.6897 | 0.892189 | 0.000218 |
| NLGN1 | 0.778983 | 0.683509 | 0.887793 | 0.000181 |
| HLA-DRA | 0.844226 | 0.748093 | 0.952712 | 0.006045 |
| CD14 | 1.167471 | 1.001541 | 1.360892 | 0.047743 |
| SALL1 | 0.674728 | 0.601662 | 0.756668 | 0 |
| EHBP1 | 0.732335 | 0.643331 | 0.833653 | 2.00E-06 |
| ITGB3 | 0.833544 | 0.714267 | 0.97274 | 0.020847 |
| TM4SF18 | 0.748148 | 0.675353 | 0.82879 | 0 |
| TMCC1 | 0.831396 | 0.741459 | 0.932243 | 0.001572 |
| VCAM1 | 0.921065 | 0.849965 | 0.998112 | 0.044849 |
| TNFRSF11B | 0.832218 | 0.748112 | 0.92578 | 0.000728 |
| CDH6 | 0.887422 | 0.807224 | 0.975586 | 0.013458 |
| TRIM22 | 0.837324 | 0.727936 | 0.963149 | 0.012932 |
| CD44 | 1.237594 | 1.069663 | 1.431888 | 0.004169 |
| CD302 | 0.761277 | 0.654165 | 0.885927 | 0.000423 |
| VWA1 | 0.758291 | 0.675053 | 0.851792 | 3.00E-06 |
| AMD1 | 0.588711 | 0.501612 | 0.690936 | 0 |
| SMIM3 | 1.155674 | 1.038194 | 1.286448 | 0.008163 |
| JUN | 0.865677 | 0.751058 | 0.997788 | 0.046533 |
| SMAD4 | 0.686602 | 0.599947 | 0.785773 | 0 |
| IFIT1 | 0.738788 | 0.645982 | 0.844927 | 1.00E-05 |
| TYMP | 1.250171 | 1.058068 | 1.477153 | 0.008714 |
| RGL1 | 0.734619 | 0.647835 | 0.833028 | 2.00E-06 |
| PLSCR4 | 0.804562 | 0.709638 | 0.912183 | 0.000686 |
| GIMAP6 | 0.700525 | 0.620127 | 0.791347 | 0 |
| P3H2 | 0.881147 | 0.778334 | 0.997539 | 0.045621 |
| GALNT14 | 0.913666 | 0.834818 | 0.999961 | 0.049902 |
| BCL9L | 0.843518 | 0.736823 | 0.965663 | 0.01365 |
| ETS1 | 0.77484 | 0.690347 | 0.869674 | 1.50E-05 |
| EPHA2 | 0.817928 | 0.710179 | 0.942025 | 0.005293 |
| GLUL | 0.833664 | 0.723404 | 0.960729 | 0.011955 |
| PLAUR | 1.526871 | 1.31771 | 1.769232 | 0 |
| HLA-DPB1 | 0.84852 | 0.751848 | 0.957622 | 0.007777 |
| AKAP12 | 0.833933 | 0.752899 | 0.92369 | 0.000498 |
| PIK3R1 | 0.755971 | 0.660169 | 0.865675 | 5.20E-05 |
| FZD1 | 0.778422 | 0.694396 | 0.872616 | 1.70E-05 |
| HERC3 | 0.77728 | 0.693162 | 0.871604 | 1.60E-05 |
| MFAP3 | 0.702857 | 0.619296 | 0.797693 | 0 |
| NPIPB12 | 1.379104 | 1.190977 | 1.596948 | 1.70E-05 |
| CXCL12 | 0.868713 | 0.775101 | 0.973632 | 0.01555 |
| THBS1 | 0.854777 | 0.761618 | 0.959332 | 0.007696 |
| PECAM1 | 0.701566 | 0.629625 | 0.781728 | 0 |
| TMEM200A | 0.818235 | 0.734623 | 0.911364 | 0.000265 |
| CITED2 | 0.781056 | 0.689961 | 0.884179 | 9.40E-05 |
| SLC16A4 | 0.799332 | 0.726693 | 0.879231 | 4.00E-06 |
| ITM2A | 0.738522 | 0.658433 | 0.828354 | 0 |
| TLN2 | 0.664259 | 0.584623 | 0.754743 | 0 |
| PROS1 | 0.85558 | 0.762675 | 0.959801 | 0.007825 |
| SOX9 | 0.883116 | 0.78793 | 0.989802 | 0.032669 |
| IFITM1 | 1.262828 | 1.072073 | 1.487525 | 0.005224 |
| FKBP10 | 1.46153 | 1.273946 | 1.676736 | 0 |
| PRSS23 | 0.727248 | 0.640036 | 0.826342 | 1.00E-06 |
| HLA-DOA | 0.88164 | 0.789992 | 0.98392 | 0.024485 |
| TMEM176B | 0.888447 | 0.817584 | 0.965452 | 0.005287 |
| NSUN5P1 | 1.381401 | 1.208525 | 1.579006 | 2.00E-06 |
| CD24 | 0.755615 | 0.661366 | 0.863295 | 3.70E-05 |
| MRC2 | 1.366907 | 1.195158 | 1.563337 | 5.00E-06 |
| TNFAIP6 | 0.906651 | 0.840431 | 0.978088 | 0.011324 |
| SLC8A1 | 0.710308 | 0.614558 | 0.820977 | 4.00E-06 |
| NEDD9 | 0.734917 | 0.652084 | 0.828271 | 0 |
| FAM110C | 0.72711 | 0.649358 | 0.814171 | 0 |
| PNMA2 | 0.832847 | 0.754265 | 0.919617 | 0.000298 |
| CTSS | 0.850312 | 0.766694 | 0.943051 | 0.002139 |
| LYVE1 | 0.802283 | 0.695592 | 0.925337 | 0.00248 |
| FIBIN | 0.82176 | 0.729365 | 0.92586 | 0.001256 |
| ABI3BP | 0.824942 | 0.7552 | 0.901123 | 2.00E-05 |
| ASMTL-AS1 | 1.291306 | 1.151003 | 1.448711 | 1.30E-05 |
| ALOX5 | 1.161127 | 1.029341 | 1.309786 | 0.01508 |
| FGL2 | 0.762927 | 0.676528 | 0.860359 | 1.00E-05 |
| GPC6 | 0.783622 | 0.69237 | 0.8869 | 0.000113 |
| UTRN | 0.746685 | 0.662045 | 0.842146 | 2.00E-06 |
| GJA1 | 0.789852 | 0.703358 | 0.886981 | 6.70E-05 |
| GUCY1A1 | 0.787063 | 0.701023 | 0.883664 | 5.00E-05 |
| CALCRL | 0.709693 | 0.636926 | 0.790774 | 0 |
| CPT1B | 1.153519 | 1.02999 | 1.291863 | 0.013463 |
| ZNF395 | 0.789199 | 0.703954 | 0.884766 | 4.90E-05 |
| EGFR | 0.840207 | 0.737851 | 0.956761 | 0.008618 |
| CD93 | 0.759522 | 0.675891 | 0.8535 | 4.00E-06 |
| TGFBR2 | 0.701895 | 0.623276 | 0.790431 | 0 |
| RHOB | 0.726835 | 0.6531 | 0.808893 | 0 |
| PDE6B | 0.823132 | 0.722193 | 0.938179 | 0.003545 |
| KRT80 | 1.164764 | 1.021617 | 1.327967 | 0.02263 |
| APOL1 | 1.132177 | 1.020158 | 1.256496 | 0.019522 |
| MXRA7 | 0.875957 | 0.773707 | 0.99172 | 0.036505 |
| F8 | 0.776961 | 0.672164 | 0.898098 | 0.000641 |
| DUSP1 | 0.765505 | 0.671957 | 0.872076 | 5.90E-05 |
| DDIT4L | 0.834659 | 0.752995 | 0.92518 | 0.000581 |
| PILRB | 1.227539 | 1.094518 | 1.376728 | 0.00046 |
| HLA-DPA1 | 0.859772 | 0.766024 | 0.964992 | 0.01032 |
| AMACR | 0.840477 | 0.745883 | 0.947069 | 0.004335 |
| PLS1 | 0.659742 | 0.589066 | 0.738898 | 0 |
| EMP1 | 0.822637 | 0.725676 | 0.932555 | 0.002279 |
| PCDHGB7 | 0.878438 | 0.787782 | 0.979527 | 0.019691 |
| MT2A | 1.256126 | 1.129126 | 1.397411 | 2.80E-05 |
| TIMP1 | 1.498051 | 1.299575 | 1.72684 | 0 |
| TSC22D3 | 0.833002 | 0.73071 | 0.949614 | 0.006269 |
| PGBD5 | 0.815253 | 0.719228 | 0.924098 | 0.001401 |
| HAVCR1 | 0.897807 | 0.834962 | 0.965382 | 0.003597 |
| GPR34 | 0.823758 | 0.720943 | 0.941237 | 0.004368 |
| TREM1 | 1.298341 | 1.162131 | 1.450517 | 4.00E-06 |
| FOLR1 | 0.867122 | 0.796385 | 0.944142 | 0.001024 |
| CTHRC1 | 1.207992 | 1.096146 | 1.33125 | 0.000138 |
| NETO2 | 0.838398 | 0.743807 | 0.945018 | 0.003904 |
| NR1H4 | 0.857386 | 0.768965 | 0.955974 | 0.005593 |
| F2RL1 | 0.715307 | 0.64406 | 0.794435 | 0 |
| ITGB8 | 0.81487 | 0.725143 | 0.915701 | 0.000583 |
| NEAT1 | 1.123364 | 1.013403 | 1.245257 | 0.026878 |
| CCND1 | 0.784548 | 0.704166 | 0.874105 | 1.10E-05 |
| SLC1A1 | 0.735102 | 0.666019 | 0.811352 | 0 |
| PODXL | 0.702635 | 0.63254 | 0.780498 | 0 |
| TSPAN18 | 0.768186 | 0.688611 | 0.856955 | 2.00E-06 |
| FMOD | 1.130212 | 1.023144 | 1.248485 | 0.015928 |
| HAGHL | 1.136223 | 1.015578 | 1.271199 | 0.025756 |
| C1S | 1.299118 | 1.170792 | 1.441508 | 1.00E-06 |
| IER3 | 1.151128 | 1.025593 | 1.292029 | 0.016899 |
| GALNT9 | 0.896788 | 0.81702 | 0.984344 | 0.021908 |
| CHI3L2 | 1.275188 | 1.158989 | 1.403037 | 1.00E-06 |
| TMEM37 | 0.786983 | 0.719655 | 0.860611 | 0 |
| FOLH1 | 0.864689 | 0.764016 | 0.978627 | 0.021332 |
| TEK | 0.621163 | 0.548038 | 0.704046 | 0 |
| ADGRL4 | 0.721524 | 0.645005 | 0.807119 | 0 |
| DCDC2 | 0.870715 | 0.771405 | 0.98281 | 0.025052 |
| APLNR | 0.748114 | 0.673631 | 0.830832 | 0 |
| COL23A1 | 0.880671 | 0.81688 | 0.949444 | 0.000925 |
| ENPEP | 0.83724 | 0.772653 | 0.907226 | 1.40E-05 |
| TIPARP | 0.83066 | 0.72402 | 0.953007 | 0.008132 |
| CD34 | 0.717648 | 0.637913 | 0.807351 | 0 |
| CDH13 | 0.731246 | 0.658571 | 0.811941 | 0 |
| TMEM176A | 0.905965 | 0.835611 | 0.982242 | 0.016648 |
| FMO2 | 0.83304 | 0.761711 | 0.911049 | 6.30E-05 |
| ERRFI1 | 0.887736 | 0.805752 | 0.978063 | 0.016012 |
| CFB | 1.2512 | 1.099053 | 1.424408 | 0.000705 |
| SCD5 | 0.751307 | 0.671531 | 0.840559 | 1.00E-06 |
| LYZ | 0.88082 | 0.810332 | 0.957439 | 0.002864 |
| SEMA5B | 0.877471 | 0.801174 | 0.961034 | 0.004858 |
| HLA-G | 0.922428 | 0.860925 | 0.988324 | 0.021815 |
| COL15A1 | 0.808677 | 0.718228 | 0.910517 | 0.00045 |
| KMO | 1.110258 | 1.002728 | 1.229319 | 0.04418 |
| SERPINF1 | 1.357261 | 1.209378 | 1.523227 | 0 |
| S1PR1 | 0.699969 | 0.628999 | 0.778947 | 0 |
| PIGY | 0.823076 | 0.754824 | 0.8975 | 1.00E-05 |
| EDN1 | 0.822571 | 0.748057 | 0.904507 | 5.50E-05 |
| KCNJ16 | 0.817035 | 0.749269 | 0.890929 | 5.00E-06 |
| AOC1 | 0.894122 | 0.8468 | 0.944089 | 5.50E-05 |
| SNURF | 0.81961 | 0.746125 | 0.900333 | 3.30E-05 |
| IGFBP5 | 0.889863 | 0.803177 | 0.985904 | 0.025652 |
| NTM | 1.171712 | 1.049695 | 1.307912 | 0.004737 |
| TGFBI | 1.138105 | 1.066015 | 1.215071 | 0.000107 |
| CYP1B1 | 1.138828 | 1.026705 | 1.263195 | 0.013958 |
| FLT1 | 0.774611 | 0.705494 | 0.850501 | 0 |
| PLIN2 | 0.865911 | 0.798055 | 0.939537 | 0.000544 |
| SERPINE1 | 1.107474 | 1.013953 | 1.209621 | 0.023342 |
| HLA-DQB2 | 0.845715 | 0.760622 | 0.940327 | 0.001954 |
| SOCS3 | 1.218536 | 1.084475 | 1.369169 | 0.000888 |
| PTPRB | 0.657954 | 0.590855 | 0.732673 | 0 |
| CDCP1 | 1.17415 | 1.051086 | 1.311622 | 0.004484 |
| UGT2A3 | 0.838983 | 0.786012 | 0.895524 | 0 |
| MAOB | 0.84673 | 0.781009 | 0.917981 | 5.40E-05 |
| MT1F | 1.177826 | 1.065629 | 1.301835 | 0.001353 |
| COL6A3 | 1.207154 | 1.085903 | 1.341944 | 0.000491 |
| MUC20 | 0.799366 | 0.726893 | 0.879064 | 4.00E-06 |
| C2 | 1.123446 | 1.0269 | 1.229069 | 0.011118 |
| FBXL16 | 0.814084 | 0.752745 | 0.880422 | 0 |
| PCSK6 | 0.900068 | 0.82558 | 0.981276 | 0.016903 |
| ZACN | 1.243999 | 1.13531 | 1.363094 | 3.00E-06 |
| IL1R2 | 1.152629 | 1.067216 | 1.244878 | 0.000299 |
| KDR | 0.738723 | 0.673606 | 0.810136 | 0 |
| TMEM252 | 0.770955 | 0.699384 | 0.849851 | 0 |
| MXRA8 | 1.238999 | 1.116218 | 1.375284 | 5.70E-05 |
| HLA-DQA2 | 0.921053 | 0.85315 | 0.99436 | 0.035316 |
| FHL1 | 0.905541 | 0.820165 | 0.999805 | 0.04955 |
| C6orf223 | 0.83086 | 0.757097 | 0.911808 | 9.40E-05 |
| AIF1L | 0.820688 | 0.7553 | 0.891737 | 3.00E-06 |
| FLRT3 | 0.738356 | 0.669518 | 0.814271 | 0 |
| MT1X | 1.159453 | 1.066858 | 1.260084 | 0.000494 |
| PLTP | 1.299953 | 1.182027 | 1.429644 | 0 |
| UGT2B7 | 0.820697 | 0.763123 | 0.882615 | 0 |
| MDK | 1.305199 | 1.183952 | 1.438862 | 0 |
| ANPEP | 0.896291 | 0.82627 | 0.972247 | 0.008336 |
| BHMT | 0.856548 | 0.804499 | 0.911963 | 1.00E-06 |
| VIL1 | 0.793958 | 0.724645 | 0.869902 | 1.00E-06 |
| GGT1 | 0.893939 | 0.827276 | 0.965973 | 0.004575 |
| SLC3A1 | 0.779773 | 0.730066 | 0.832865 | 0 |
| APOLD1 | 0.733479 | 0.668072 | 0.805289 | 0 |
| SLC44A4 | 0.752754 | 0.673064 | 0.84188 | 1.00E-06 |
| ACMSD | 0.851471 | 0.788813 | 0.919105 | 3.70E-05 |
| TSPAN1 | 0.833627 | 0.771718 | 0.900502 | 4.00E-06 |
| CUBN | 0.795587 | 0.742308 | 0.85269 | 0 |
| CLDN2 | 0.891532 | 0.836081 | 0.950661 | 0.000458 |
| COL1A1 | 1.153557 | 1.057611 | 1.258206 | 0.001263 |
| SFRP2 | 1.084752 | 1.01758 | 1.156358 | 0.012621 |
| ENPP3 | 0.901451 | 0.842927 | 0.964038 | 0.002451 |
| SLC34A2 | 1.066736 | 1.000343 | 1.137535 | 0.04879 |
| MMP7 | 1.069334 | 1.004717 | 1.138106 | 0.035035 |
| SPON1 | 0.92252 | 0.860126 | 0.989441 | 0.024005 |
| LRP2 | 0.850726 | 0.800525 | 0.904075 | 0 |
| LUM | 1.086767 | 1.018393 | 1.15973 | 0.012083 |
| GSTA1 | 0.926522 | 0.876847 | 0.979012 | 0.00664 |
| SLPI | 1.128913 | 1.073607 | 1.187068 | 2.00E-06 |
| CXCL14 | 0.900887 | 0.844298 | 0.961269 | 0.001614 |
| IGHA1 | 1.083219 | 1.013332 | 1.157926 | 0.018815 |
| C1QL1 | 1.130731 | 1.049159 | 1.218645 | 0.001299 |
| PAH | 0.868852 | 0.804628 | 0.938203 | 0.000333 |
| PTHLH | 1.081565 | 1.014806 | 1.152716 | 0.015862 |
| LTF | 0.920832 | 0.852978 | 0.994085 | 0.034698 |
| NPTX2 | 1.078499 | 1.008806 | 1.153006 | 0.02661 |
| KRT19 | 1.088917 | 1.029944 | 1.151267 | 0.002713 |
| IGHG1 | 1.099486 | 1.042521 | 1.159563 | 0.000476 |
