# Supplementary figures and images for: Identification of Prognostic Metabolism-Related Genes in Clear Cell Renal Cell Carcinoma
Source: J Oncol. 2021 Sep 27;2021:2042114. doi: 10.1155/2021/2042114 (PMC8490028; doi:10.1155/2021/2042114)

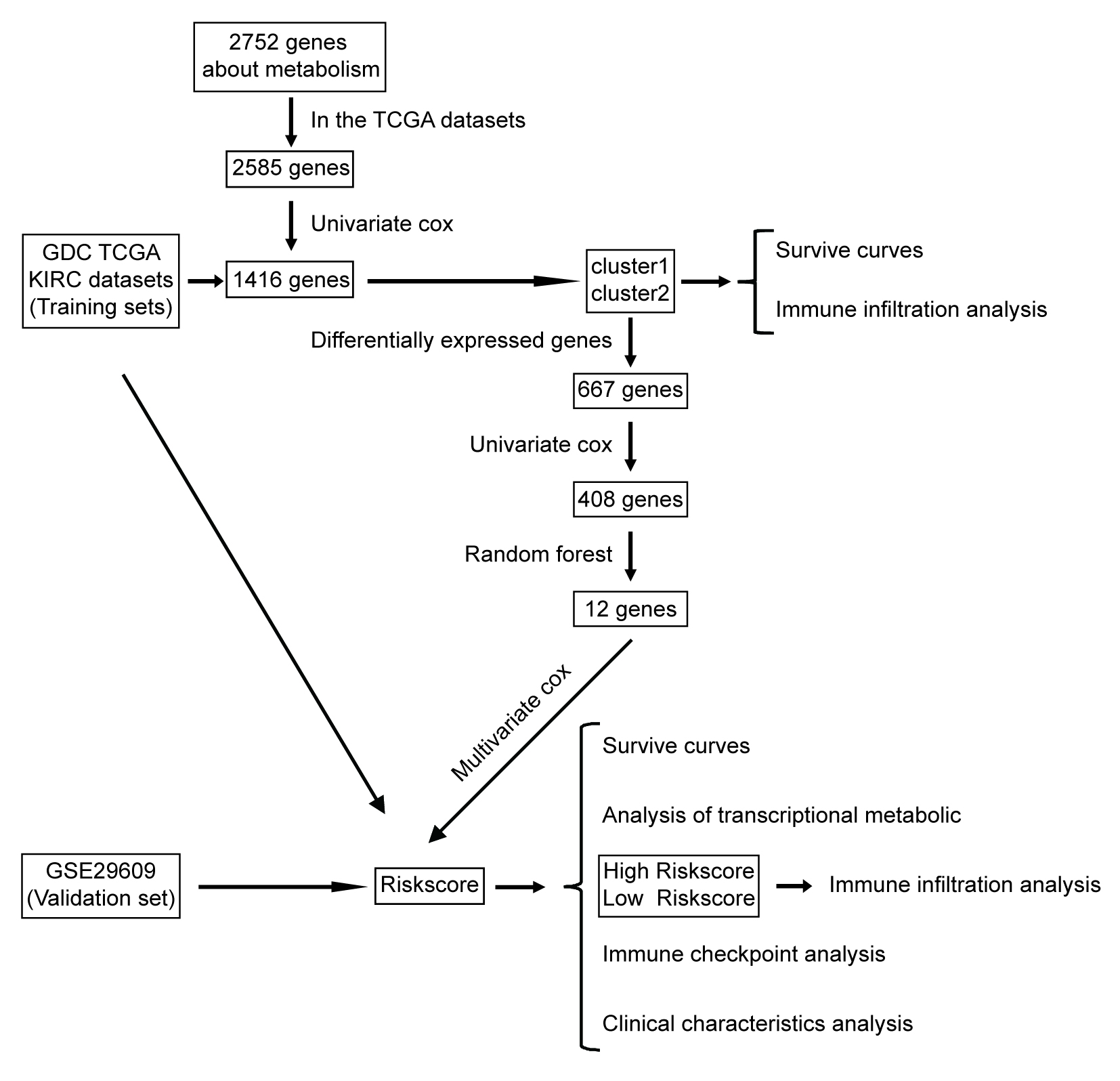

Supplement: Supplementary Materials — Supplementary Table S1: clinicopathologic parameters of TCGA KIRC dataset. Supplementary Table S2: DEG to clusters. Supplementary Table S3: univar result after limma final. Supplementary Figure S1: flowchart of data collection and analysis in this study. Supplementary Figure S2: the relationship between the cophenetic coefficient and the number of clusters. Supplementary Figure S3: survival analysis of MDK, SGCB, C4orf3, PILRB, IGHG1, IFITM1, MUC20, and KRT80. [file 2042114.f1.zip › 2042114.f1/Supplementary Figure S1.jpg]

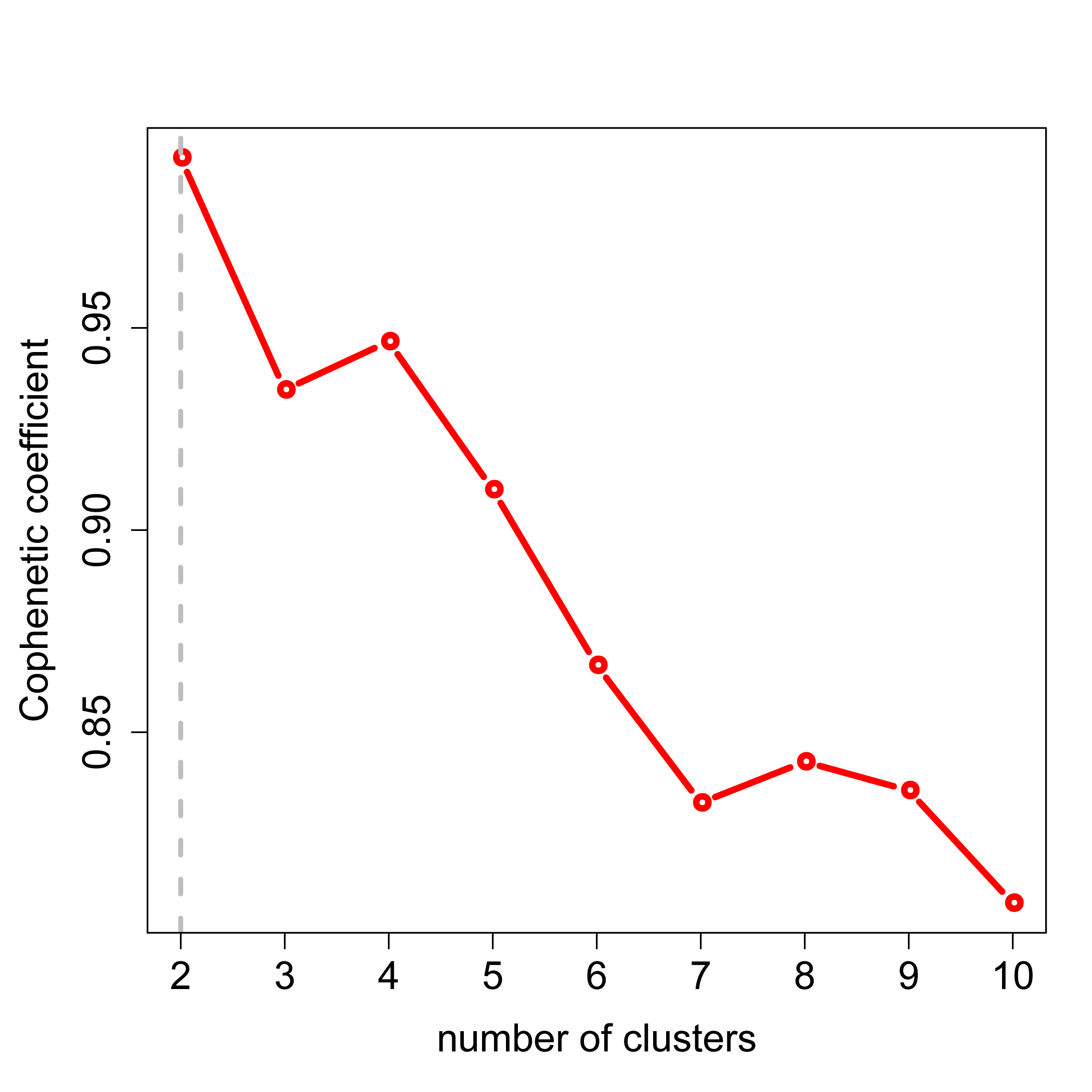

Supplement: Supplementary Materials — Supplementary Table S1: clinicopathologic parameters of TCGA KIRC dataset. Supplementary Table S2: DEG to clusters. Supplementary Table S3: univar result after limma final. Supplementary Figure S1: flowchart of data collection and analysis in this study. Supplementary Figure S2: the relationship between the cophenetic coefficient and the number of clusters. Supplementary Figure S3: survival analysis of MDK, SGCB, C4orf3, PILRB, IGHG1, IFITM1, MUC20, and KRT80. [file 2042114.f1.zip › 2042114.f1/Supplementary Figure S2.jpg]

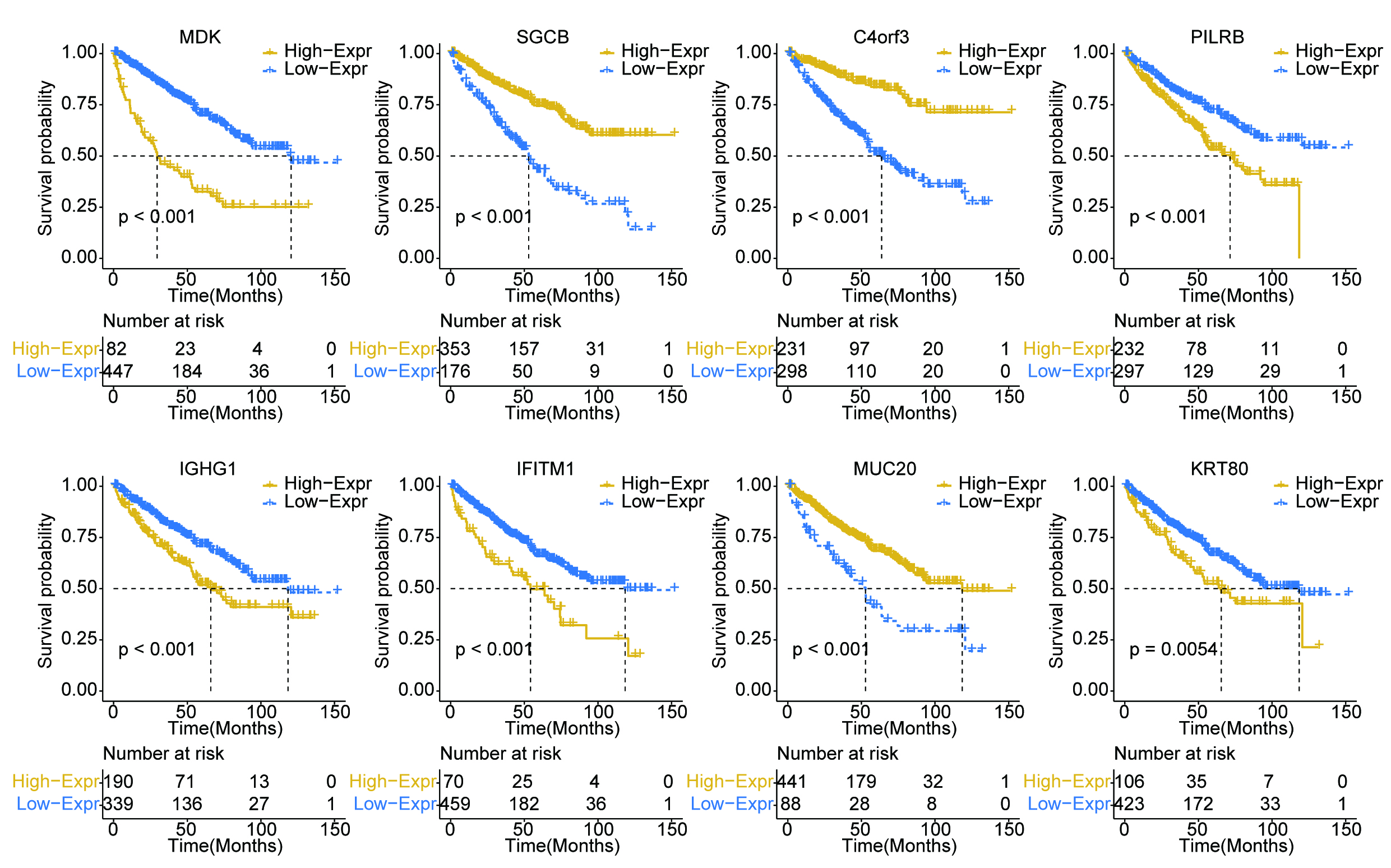

Supplement: Supplementary Materials — Supplementary Table S1: clinicopathologic parameters of TCGA KIRC dataset. Supplementary Table S2: DEG to clusters. Supplementary Table S3: univar result after limma final. Supplementary Figure S1: flowchart of data collection and analysis in this study. Supplementary Figure S2: the relationship between the cophenetic coefficient and the number of clusters. Supplementary Figure S3: survival analysis of MDK, SGCB, C4orf3, PILRB, IGHG1, IFITM1, MUC20, and KRT80. [file 2042114.f1.zip › 2042114.f1/Supplementary Figure S3.jpg]
